# Long-Term Impact of Clinical Obesity on Heart Failure Development and Prognosis: New Insights

**DOI:** 10.1016/j.jacasi.2026.01.031

**Published:** 2026-05-02

**Authors:** Hongmin Liu, Tong Liu, Yuntao Wu, Haiyan Zhao, Liming Lin, Xiang Gao, Shouling Wu

**Affiliations:** a Department of Cardiology, Kailuan General Hospital, Tangshan, China; b Tianjin Key Laboratory of Ionic-Molecular Function of Cardiovascular Disease, Department of Cardiology, Tianjin Institute of Cardiology, the Second Hospital of Tianjin Medical University, Tianjin, China; c Department of Nutrition and Food Hygiene, School of Public Health, Institute of Nutrition, Fudan University, Shanghai, China

**Keywords:** all-cause mortality, clinical obesity, heart failure, risk prediction

## Abstract

**Background:**

*The Lancet Diabetes & Endocrinology* Commission recognizes clinical obesity as an independent disease, although its link to heart failure (HF) remains underexplored.

**Objectives:**

The goal of this study was to evaluate the impact of clinical obesity on new-onset HF risk and subsequent all-cause mortality after the development of HF.

**Methods:**

Clinical obesity was defined based on body mass index, waist circumference, waist-to-hip ratio, waist-to-height ratio, and 10 clinical criteria. A total of 99,131 participants from the Kailuan Study cohort were classified into nonobese, preclinical obesity, and clinical obesity groups. A Cox proportional hazards model was applied to assess the association between clinical obesity and the risk of new-onset HF, and all-cause mortality was examined among those with HF.

**Results:**

Over a median follow-up of 16.0 years, 3,280 participants developed HF, and there were 19,170 deaths from any cause. Compared with the nonobese group, clinical obesity was associated with a 63% higher risk of new-onset HF (adjusted [aHR]: 1.63; 95% CI: 1.49-1.77), with varying risks across HF subtypes. Within the clinical obesity group, HF risk increased with the number of clinical criteria: one (aHR: 1.50; 95% CI: 1.37-1.64), two (aHR: 1.91; 95% CI: 1.70-2.14), and three or more (aHR: 2.20; 95% CI: 1.80-2.68). However, clinical obesity was not associated with increased all-cause mortality after HF (aHR: 1.01; 95% CI: 0.90-1.15).

**Conclusions:**

These findings highlight clinical obesity as a risk factor for new-onset HF and all-cause mortality, driven both by excess weight and associated clinical conditions. Recognizing clinical obesity as an independent disease may support more effective HF prevention strategies.

With societal progress, the widespread availability of food (particularly highly processed and calorie-dense options), coupled with a decline in physical activity that contributes to energy expenditure, has driven a rapid global increase in body weight. The prevalence of overweight and obesity has increased significantly, rising from 25% and 6% in 1990 to 43% and 16% in 2022, respectively, with global obesity rates more than doubling over this period.^1^ This trend has been accompanied by a corresponding rise in the prevalence of chronic non-communicable diseases linked to overweight and obesity. For example, the worldwide prevalence of heart failure (HF) increased by 29% between 2010 and 2019, with HF with preserved ejection fraction (HFpEF) now accounting for approximately one-half of all HF cases in the United States and up to 65.4% in Portugal, placing a substantial burden on the global economy.^2^ As a result, overweight and obesity have emerged as critical targets for intervention in the prevention and management of chronic non-communicable diseases. In response, drugs specifically aimed at weight reduction, such as glucagon-like peptide-1 receptor agonists, have been developed. In addition, management guidelines for overweight and obesity have been issued by Europe, the United States, and China.^3^, ^4^, ^5^, ^6^

An effective intervention target requires a precise definition, which is a cornerstone of precision medicine. The World Health Organization recognized obesity as a disease as early as 1948 and assigned it a disease code (International Classification of Diseases, 10th Revision, E66) in 1997.^7^^,^^8^ However, although body mass index (BMI) is commonly used to diagnose obesity, it fails to accurately reflect its underlying pathophysiology, excessive fat accumulation. Waist circumference and waist-to-hip ratio, although alternative measures, also do not fully capture body fat distribution or the clinical implications of obesity. Relying solely on BMI for the diagnosis of obesity impedes accurate diagnosis and decision-making for intervention, including surgical and pharmacologic treatments.

To overcome the limitations and shortcomings of traditional obesity diagnosis and achieve more accurate assessments, *The Lancet Diabetes & Endocrinology* Commission has proposed the “Definition and Diagnostic Criteria for Clinical Obesity.”^9^ This new standard not only requires at least 2 anthropometric indicators to meet the criteria for obesity but also incorporates 18 clinical criteria related to the condition. Compared with traditional diagnostic standards, this framework offers a more precise and comprehensive approach to obesity diagnosis.

Among the 18 clinical criteria for clinical obesity, HF is situated downstream in the cardiovascular event cascade, representing an irreversible endpoint. In contrast, hypertension, metabolic disturbances in glucose and lipids, and sleep apnea are positioned upstream in this chain. Previous studies have shown that these factors elevate the risk of developing HF and are positively associated with adverse outcomes in individuals with HF.^10^, ^11^, ^12^ To our knowledge, no research has yet explored the impact of the new diagnostic criteria on HF. To fill this gap, we analyzed data from the Kailuan Study cohort to assess the influence of these updated criteria on the incidence of new-onset HF.

## Methods

### Study Design and Participants

This study is based on data analysis from the Kailuan Study, a research project focused on cardiovascular and cerebrovascular diseases, along with associated risk factors and interventions in China.^13^ The Kailuan General Hospital and its 11 affiliated hospitals provide routine medical services to both active and retired employees. All employees are enrolled in social and medical insurance programs. The study started in 2006 and began with an initial health examination of 101,510 employees of the Kailuan Group Co, Ltd. Information was obtained through questionnaires, including demographic data, lifestyle and habits (eg, physical activity, snoring), health status (eg, history of diabetes, hypertension, chronic obstructive pulmonary disease, sleep apnea, menstrual history, pregnancy and childbirth history, medication history), clinical physical examination (eg, height, weight, waist circumference, hip circumference, blood pressure), and laboratory tests (eg, fasting blood glucose, lipid profile, serum creatinine, urinalysis). Annual discharge records were also reviewed to monitor the occurrence of events such as atrial fibrillation, HF, chronic obstructive pulmonary disease, sleep apnea syndrome, urinary incontinence, deep vein thrombosis/pulmonary embolism, hip/knee osteoarthritis, pulmonary hypertension, and intracranial hypertension. In addition, under medical insurance regulations, individuals diagnosed with any of the 93 chronic diseases (Supplemental Table 3 in the Supplemental Material) covered by the insurance are eligible for specialized outpatient care with enhanced reimbursement rates, with diagnoses confirmed by expert panels in the relevant specialties. These data provide the foundation for the current analysis of clinical obesity.

The following individuals met inclusion criteria: 1) those who participated in both the baseline and at least one follow-up assessment; 2) those with data on BMI, waist circumference, waist-to-hip ratio, and waist-to-height ratio that could be used to assess obesity status; 3) those with sufficient clinical information to assess whether they exhibited signs or symptoms of ongoing obesity-related organ system dysfunction; and 4) participants who provided signed informed consent and agreed to participate in the study.

Individuals with the following were excluded: 1) Cushing’s syndrome; 2) chronic glomerulonephritis; 3) autoimmune diseases (rheumatoid arthritis, systemic lupus erythematosus, Sjögren's syndrome); and 4) a history of HF at baseline.

This study was conducted in accordance with the principles of the Declaration of Helsinki and received approval from the Ethics Committee of the Kailuan Medical Group (approval no. 2006 Medical Ethics 5).

### Data Collection

#### Collection of General Information

Basic demographic data, lifestyle and behavioral habits, and health status (including medical history, chronic disease conditions, and medication use) were gathered through structured questionnaires. Further details have been given in previously published literature.^13^

Anthropometric and laboratory measurements were collected (Supplemental Appendix A). Relevant definitions are provided in Supplemental Appendix B.

#### Definition of Clinical Obesity

In accordance with recommendations from *The Lancet Diabetes & Endocrinology* Commission, the diagnosis of clinical obesity requires the fulfillment of the following criteria (Supplemental Appendix C): 1) confirmation of obesity status through anthropometric measurements (Supplemental Table 1); and 2) meeting at least one clinical criterion (Supplemental Table 2).

Regarding the 18 diagnostic criteria for clinical obesity proposed by the commission, certain limitations in data sources have resulted in the omission of information pertaining to the male and female reproductive systems and the lymphatic system. Although data on increased intracranial pressure, non-alcoholic fatty liver disease with hepatic fibrosis, and activity levels derived from the activities of daily living questionnaire became available after follow-up, baseline data for the entire cohort from 2006 were unavailable. As a result, these 6 indicators were excluded. HF, being the primary endpoint of the study, is therefore considered within the 10 diagnostic criteria included, which are as follows: hypertension, metabolic dysfunction, upper airway disorders (sleep apnea/chronic snoring), respiratory conditions (chronic obstructive pulmonary disease), atrial fibrillation, pulmonary artery hypertension, deep vein thrombosis and/or pulmonary thromboembolic disease, renal injury, recurrent/chronic urinary incontinence, and knee or hip osteoarthritis. The diagnostic information for chronic diseases from outpatient records is provided in Supplemental Table 3.

#### Definition of Preclinical Obesity

Preclinical and clinical obesity are diagnosed using the same anthropometric thresholds to confirm obesity status; however, preclinical obesity does not meet any clinical criteria for obesity-related conditions.

All participants were classified into nonobese, preclinical obesity, and clinical obesity groups according to the diagnostic criteria for clinical obesity.

#### Definition and Stratification of Covariates

The specific stratification and definition of covariates are provided in Supplemental Table 4. All variables were collected at baseline between 2006 and 2007, with missing data accounting for <5%, as shown in Supplemental Table 5.

#### Endpoint Events

The primary endpoint events were defined as HF or death. HF was diagnosed by clinicians using standardized criteria, which included symptoms, clinical signs, N-terminal pro-B-type natriuretic peptide levels, and echocardiographic findings. The diagnostic criteria for HF in this study were based on the Chinese Guidelines for the Diagnosis and Treatment of Chronic Heart Failure (2007),^14^ consistent with the standards at the time the Kailuan Study began. The criteria were also aligned with the 2016 European Society of Cardiology Guidelines for the Diagnosis and Treatment of Acute and Chronic Heart Failure during the follow-up period.^15^

A diagnosis of HF required the fulfillment of criterion 1 and at least one of the following criterion 2 or criterion 3: 1) symptoms and signs of heart failure, including dyspnea, fatigue, palpitations, fluid retention, and a NYHA functional class of II or higher; 2) left ventricular ejection fraction (LVEF) <50%; and 3) plasma N-terminal pro-B-type natriuretic peptide level ≥125 ng/L.

All events were coded according to the International Classification of Diseases-10th Revision, with heart failure coded as I50.907, I50.908, I50.909, and I50.910, based on detailed diagnostic information obtained through medical record review. Mortality data were obtained from the Kailuan Social Security Registration System. Follow-up for HF events continued until December 31, 2022, and for mortality until December 31, 2023. Participants without endpoint events were followed up until December 31, 2022.

LVEF was assessed as described in Supplemental Appendix D. HF was classified based on LVEF into 3 categories: HFpEF, HF with mid-range ejection fraction (HFmrEF), and HF with reduced ejection fraction (HFrEF). HFrEF is identified in patients with an LVEF <40%, HFmrEF is diagnosed in patients with an LVEF of 40% to 49% and HFpEF is categorized as an LVEF ≥50%.

### Statistical Analysis

Normality of all baseline characteristic variables was assessed. Continuous variables with a normal distribution are described as mean ± SD and were analyzed by using one-way analysis of variance. Continuous variables with a skewed distribution are described as median (Q1-Q3) and were analyzed by using the Kruskal-Wallis test. Categorical variables are expressed as proportions and were analyzed by using the chi-square test. The Kaplan-Meier method was used to estimate the cumulative incidence of new-onset HF events, with comparisons between groups made using the log-rank test.

Cox proportional hazards models were used to examine the effect of clinical obesity on the incidence of new-onset HF, with nonobese individuals as the reference group. To examine the separate and combined associations of obesity and clinical criteria with the risk of HF and post-HF all-cause mortality, the nonobese group was further stratified based on the presence of clinical criteria or overweight status.

The details of Models 1, 2, and 3 are provided in Supplemental Appendix E.

We further analyzed whether there is a dose–response relationship between the number of clinical criteria for clinical obesity and the risk of HF, specifically examining the impact of 1, 2, or ≥3 clinical criteria on the risk of HF. In addition, we assessed the effect of clinical obesity on the subtypes of HF, including HFpEF, HFmrEF, HFrEF, and unclassified HF.

### Sensitivity and Robustness Analysis

To assess robustness, sensitivity analyses were conducted by excluding participants with less than two years of follow-up or a history of myocardial infarction or cancer, to evaluate their impact on the obesity/HF relationship. Mortality as a competing risk was accounted for by using the Fine and Gray subdistribution hazard model.

To address potential confounding, sensitivity analyses using propensity score matching were performed to establish a balanced cohort between the clinically obese and nonobese groups. Specific details of the matching procedure are provided Supplemental Appendix F.

To validate the representativeness of the study population, BMI was used as a measure of obesity, and the impact of traditional obesity indicators on HF and its prognosis was analyzed.

### Subgroup Analysis

To explore the moderating effect of covariates on the relationship between clinical obesity and new-onset HF, we first conducted interaction analyses by including each covariate as an interaction term in the regression models with the independent variable. Covariates with significant interaction effects were further analyzed in subgroup analyses.

### Prognostic Analysis of HF

We further analyzed the impact of clinical obesity on all-cause mortality following the onset of HF. All-cause mortality was considered a survival event in HF. Survival curves illustrated differences among the 3 groups. The effect of clinical obesity on the prognosis of patients with HF was assessed by using Cox proportional hazards models.

### Exploratory Analyses

To explore potential causal pathways linking obesity, obesity-related clinical complications (clinical criteria), and HF, mediation analyses were performed to determine whether obesity mediated the association between clinical complications and HF, or vice versa. Because both obesity and obesity-related clinical complications (clinical criteria) were assessed concurrently at baseline, the temporal sequence between these 2 variables could not be established. To address this issue, 2 exploratory mediation analyses were conducted under alternative causal assumptions. In Pathway A, obesity was considered the exposure, clinical criteria the mediator, and HF the outcome (obesity [X] → clinical criteria [M] → HF [Y]). In Pathway B, the temporal order was reversed (clinical criteria [X] → obesity [M] → HF [Y]).

To clarify the contribution of obesity vs relevant comorbid diseases (clinical criteria), a Cox regression analysis was performed with obesity and clinical criteria as covariates. The population attributable risk percentage (PAR%) was calculated for each factor.

Given the overlap between clinical obesity and the definitions of metabolic syndrome, a comparative analysis was also conducted. The definition of metabolic syndrome is provided in Supplemental Appendix G.

Statistical analyses were conducted by using SAS software version 9.4 (SAS Institute, Inc). All tests were 2-sided, and a *P* value of <0.05 was considered statistically significant.

## Results

### Baseline Characteristics

A total of 99,367 participants met the inclusion criteria. After excluding 139 individuals with Cushing’s syndrome, glomerulonephritis, rheumatoid arthritis, systemic lupus erythematosus, or Sjögren's syndrome, and 97 individuals with a history of HF, 99,131 participants were included in the final analysis (Supplemental Figure 1). The baseline characteristics are presented in accordance with the Strengthening the Reporting of Observational Studies in Epidemiology guidelines.^16^^,^^17^ The baseline age was 51.76 ± 12.57 years, and there were 79,218 male patients (79.9%) and 19,913 female patients (20.1%). The nonobese group, preclinical obesity group, and clinical obesity group comprised 45,264 (45.7%), 18,977 (19.1%), and 34,890 (35.2%) individuals, respectively.

Compared with the nonobese group, the clinical obesity group had the highest proportions of salt intake >12 g. In addition, the clinical obesity group exhibited higher systolic blood pressure, diastolic blood pressure, heart rate, uric acid, fasting blood glucose, high-sensitivity C-reactive protein, and triglyceride levels and a lower estimated glomerular filtration rate (all, *P* < 0.001). Furthermore, the clinical obesity group had the highest rates of myocardial infarction, stroke, diabetes, hypertension, and use of antihypertensive, antidiabetic, and lipid-lowering medications (Table 1).

**Table 1:** Baseline Characteristics of All Participants Stratified According to Obesity Status

	Total (N = 99,131)	Nonobese (n = 45,264)	Preclinical Obesity (n = 18,977)	Clinical Obesity (n = 34,890)
Age, y	51.76 ± 12.57	49.29 ± 12.86	50.11 ± 11.89	55.86 ± 11.46
Male	79,218 (79.9)	35,901 (79.3)	14,143 (74.5)	29,174 (83.6)
Smoking status				
Never	57,757 (59.5)	25,925 (57.9)	12,513 (66.0)	19,319 (57.9)
Former	5,759 (5.93)	2,229 (4.98)	814 (4.30)	2,716 (8.14)
Occasional	3,476 (3.58)	1,759 (3.93)	665 (3.51)	1,052 (3.15)
Current	30,057 (31.0)	14,829 (33.1)	4,956 (26.2)	10,272 (30.8)
Drinking status				
Non-drinker	56,833 (58.5)	25,370 (56.7)	12,275 (64.8)	19,188 (57.5)
Occasional drinker	22,790 (23.5)	11,285 (25.2)	3,968 (21.0)	7,537 (22.6)
Current drinker	17,455 (18.0)	8,098 (18.1)	2,710 (14.3)	6,647 (19.9)
Physical activity				
Never	8,445 (8.78)	4,373 (9.83)	1,269 (6.71)	2,803 (8.56)
Occasionally	72,587 (75.5)	33,208 (74.6)	15,546 (82.1)	23,833 (72.8)
Regularly	15,131 (15.7)	6,920 (15.6)	2,111 (11.2)	6,100 (18.6)
Marital status				
Unmarried	1,638 (1.70)	1,256 (2.82)	245 (1.29)	137 (0.42)
Married	90,911 (94.3)	41,680 (93.5)	18,147 (95.7)	31,084 (94.6)
Divorced	840 (0.87)	427 (0.96)	152 (0.80)	261 (0.79)
Widowed	1,994 (2.07)	785 (1.76)	264 (1.39)	945 (2.88)
Remarried	1,021 (1.06)	439 (0.98)	161 (0.85)	421 (1.28)
Educational level				
No formal education	1,214 (1.26)	471 (1.06)	168 (0.89)	575 (1.75)
Primary education	9,342 (9.69)	3,798 (8.52)	1,275 (6.72)	4,269 (13.0)
Junior high school	66,460 (69.0)	29,359 (65.9)	14,163 (74.7)	22,938 (69.9)
High school education	12,687 (13.2)	6,881 (15.4)	2,191 (11.6)	3,615 (11.0)
University education or higher	6,660 (6.91)	4,057 (9.10)	1,166 (6.15)	1,437 (4.38)
Sedentary time, h/d				
<4	71,708 (74.6)	32,606 (73.3)	14,784 (78.2)	24,318 (74.4)
4-8	21,248 (22.1)	10,347 (23.3)	3,630 (19.2)	7,271 (22.2)
>8	3,126 (3.25)	1,542 (3.47)	491 (2.60)	1,093 (3.34)
Salt intake, g/d				
<6	8,964 (9.32)	4,555 (10.2)	1,399 (7.39)	3,010 (9.19)
6-12	76,819 (79.8)	35,252 (79.2)	16,002 (84.5)	25,565 (78.0)
>12	10,441 (10.9)	4,728 (10.6)	1,530 (8.08)	4,183 (12.8)
Heart rate, beats/min	73.79 ± 10.18	73.48 ± 10.20	72.70 ± 9.37	74.78 ± 10.48
Urine acid, μmol/L	290.08 ± 84.17	278.11 ± 77.55	288.53 ± 81.79	306.42 ± 90.72
BMI, kg/m^2^	25.05 ± 3.49	23.09 ± 2.56	25.88 ± 3.24	27.14 ± 3.27
Weight, kg	70.29 ± 11.38	64.89 ± 9.03	72.35 ± 11.02	76.16 ± 11.03
Height, cm	167.37 ± 6.98	167.50 ± 6.83	167.03 ± 7.17	167.40 ± 7.07
Hip circumference, cm	97.35 ± 8.87	92.89 ± 6.87	99.63 ± 8.48	101.95 ± 8.58
Waist circumference, cm	87.06 ± 10.03	79.21 ± 6.07	92.51 ± 7.64	94.27 ± 7.65
Waist-to-hip ratio	0.89 ± 0.07	0.85 ± 0.06	0.93 ± 0.07	0.93 ± 0.06
Waist-to-height ratio	0.52 ± 0.06	0.47 ± 0.03	0.55 ± 0.04	0.56 ± 0.05
Systolic blood pressure, mm Hg	131.04 ± 21.06	126.28 ± 20.10	118.98 ± 10.48	143.73 ± 20.15
Diastolic blood pressure, mm Hg	83.52 ± 11.78	81.08 ± 11.28	77.27 ± 6.48	90.04 ± 11.60
eGFR, mL/min/1.73 m^2^	80.81 (67.58–95.24)	82.18 (68.88–96.59)	84.26 (71.19–97.86)	77.00 (64.26–91.57)
Fasting blood glucose, mmol/L	5.48 ± 1.69	5.30 ± 1.46	5.21 ± 1.26	5.86 ± 2.05
hs-CRP, mg/L	0.80 (0.30-2.20)	0.60 (0.22-1.53)	0.80 (0.30-2.10)	1.22 (0.50-3.30)
LDL-C, mmol/L	2.35 ± 0.91	2.37 ± 0.84	2.24 ± 0.88	2.37 ± 1.02
HDL-C, mmol/L	1.55 ± 0.40	1.57 ± 0.40	1.52 ± 0.39	1.54 ± 0.42
Triglycerides, mmol/L	1.27 (0.89-1.93)	1.10 (0.78-1.57)	1.28 (0.92-1.86)	1.60 (1.11-2.43)
Myocardial infarction	1,271 (1.32)	426 (0.96)	145 (0.76)	700 (2.13)
Stroke	2,502 (2.59)	809 (1.81)	210 (1.11)	1,483 (4.51)
Diabetes	9,305 (9.39)	2,684 (5.93)	832 (4.38)	5,789 (16.6)
Hypertension	45,350 (45.7)	15,986 (35.3)	0	29,364 (84.2)
Antidiabetic agents	2,442 (2.53)	686 (1.54)	205 (1.08)	1,551 (4.69)
Lipid-lowering agents	942 (0.97)	290 (0.65)	66 (0.35)	586 (1.74)
Blood pressure–lowering agents	11,143 (11.4)	3,219 (7.19)	0	7,924 (23.4)

Values are mean ± SD or median (Q1-Q3), rounded to 2 decimal places. Proportional data (rates) are presented as n (%) with 3 significant figures. Estimated glomerular filtration rate (eGFR), high-sensitivity C-reactive protein (hs-CRP), and triglycerides exhibited skewed distributions and were analyzed by using the Kruskal-Wallis test.

BMI = body mass index; HDL-C = high-density lipoprotein cholesterol; LDL-C = low-density lipoprotein cholesterol.

The anthropometric measurements used for diagnosing clinical obesity are provided in Supplemental Table 6, and the distribution of clinical criteria is presented in Supplemental Table 7. In the clinically obese group, up to five clinical criteria can be simultaneously met. Among these individuals, 25,199 (72.22%) met 1 clinical criterion, 8,366 (23.98%) met 2 criteria, 1,238 (3.55%) met 3 criteria, 84 (0.24%) met 4 criteria, and 3 (0.01%) met 5 criteria. The most common clinical criterion was hypertension, observed in 29,364 (84.16%) individuals, followed by habitual snoring in 8,739 (20.05%) and metabolic dysfunction in 6,022 (17.26%). In the nonobese group, multiple clinical criteria were also present, with hypertension found in 15,986 (35.32%) individuals, followed by snoring and metabolic dysfunction in 4,941 (10.92%) and 1,944 (4.29%) individuals, respectively.

**Figure 1:**
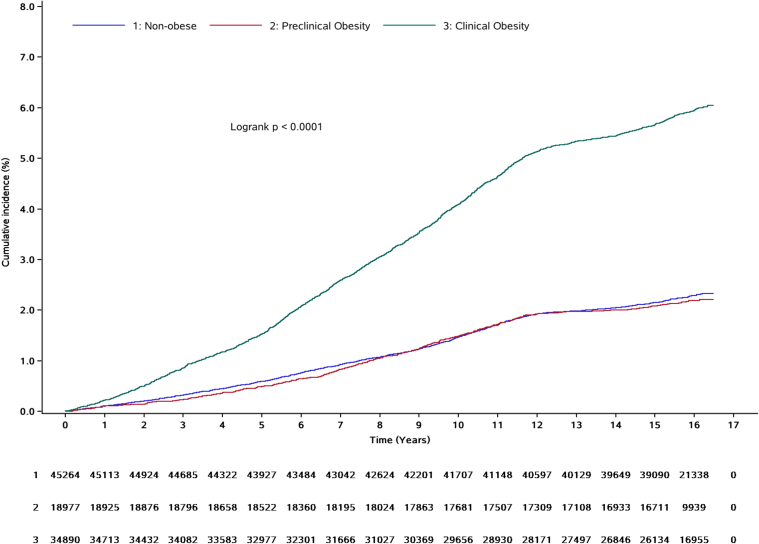
Kaplan-Meier and Cumulative Incidence Plots for Heart Failure The Kaplan-Meier method was used to estimate the cumulative incidence of new-onset heart failure events, with comparisons between groups made using the log-rank test. The clinical obesity group had the highest cumulative incidence. The log-rank test revealed a significant difference in cumulative incidence across the 3 groups.

During a median follow-up of 16.0 years (Q1-Q3: 15.6-16.2 years), 3,280 incident cases of HF (3.3%) and 19,170 deaths from any cause (19.3%) were recorded. The incidence density of HF in the nonobese group was 1.45 per 1,000 person-years (‰PY), 1.38‰PY in the preclinical obesity group, and 3.85‰PY in the clinical obesity group (Table 2). Figure 1 shows the cumulative incidence of HF by group, based on Kaplan-Meier curves. The clinical obesity group had the highest cumulative incidence. The log-rank test revealed a significant difference in cumulative incidence across the 3 groups (*P* < 0.001).

**Table 2:** Associations Between Clinical Obesity and the Risk of Heart Failure

Clinical Criteria		Events/Total	Incidence Density (per 1,000 person-years)	Model 1 aHR (95% CI)	Model 2 aHR (95% CI)	Model 3 aHR (95% CI)
Nonobese		981/45,264	1.45 (1.36-1.54)	Reference	Reference	Reference
Preclinical obesity		397/18,977	1.38 (1.25-1.52)	0.95 (0.84-1.06)	0.96 (0.85-1.08)	1.01 (0.90-1.15)
Clinical obesity		1,902/34,890	3.85 (3.68-4.03)	1.89 (1.75-2.05)	1.76 (1.62-1.92)	1.63 (1.49-1.77)
Stratification of the nonobese group into overweight and normal weight individuals based on BMI categories						
Nonobese (normal weight)	–	597/28,664	1.40 (1.29-1.51)	Reference	Reference	Reference
Nonobese (over weight)	–	367/15,843	1.53 (1.38-1.70)	1.21 (1.06-1.38)	1.17 (1.02-1.35)	1.15 (1.00-1.32)
Preclinical obesity	Absent	397/18,977	1.38 (1.25-1.52)	1.02 (0.90-1.15)	1.02 (0.90-1.17)	1.07 (0.94-1.22)
Clinical obesity	Present	1,902/34,890	3.85 (3.68-4.03)	2.04 (1.86-2.23)	1.88 (1.70-2.07)	1.72 (1.55-1.90)
Stratification of the nonobese group based on the presence of clinical criteria						
Nonobese	Absent	272/25,288	0.70 (0.62-0.79)	Reference	Reference	Reference
Nonobese	Present	709/19,976	2.45 (2.28-2.64)	2.19 (1.90-2.52)	2.06 (1.78-2.39)	1.91 (1.65-2.22)
Preclinical obesity	Absent	397/18,977	1.38 (1.25-1.52)	1.55 (1.33-1.81)	1.51 (1.29-1.77)	1.51 (1.29-1.77)
Clinical obesity	Present	1,902/34,890	3.85 (3.68-4.03)	3.15 (2.77-3.58)	2.81 (2.46-3.22)	2.52 (2.19-2.89)
Stratification of the nonobese group based on overweight status and the presence of clinical criteria						
Nonobese (normal weight)	Absent	187/17,584	0.69 (0.60-0.80)	Reference	Reference	Reference
Nonobese (normal weight)	Present	410/11,080	2.60 (2.36-2.87)	2.16 (1.82-2.57)	2.04 (1.70-2.45)	1.90 (1.58-2.28)
Nonobese (overweight)	Absent	81/7,451	0.70 (0.56-0.87)	1.09 (0.84-1.42)	1.06 (0.81-1.39)	1.05 (0.80-1.38)
Nonobese (overweight)	Present	286/8,392	2.31 (2.06-2.59)	2.41 (2.00-2.89)	2.21 (1.82-2.68)	2.03 (1.67-2.46)
Preclinical obesity	Absent	397/18,977	1.38 (1.25-1.52)	1.60 (1.35-1.91)	1.55 (1.29-1.85)	1.55 (1.29-1.85)
Clinical obesity	Present	1,902/34,890	3.85 (3.68-4.03)	3.25 (2.79-3.78)	2.88 (2.46-3.37)	2.57 (2.19-3.01)

Model 1 was adjusted for age and sex. Model 2 was further adjusted for smoking status, alcohol drinking status, physical activities, marital status, educational level, sedentary time, intake of salt, heart rate, high-sensitivity C-reactive protein, urine acid, low-density lipoprotein cholesterol, myocardial infarction, and stroke based on model 1. Model 3 was further adjusted for blood pressure–lowering agents, lipid-lowering agents, and antidiabetic agents based on model 2.

Table 2 presents the association between clinical obesity and the risk of new-onset HF. After adjusting for covariates, the aHRs for the preclinical and clinical obesity groups were 1.01 (95% CI: 0.90-1.15) and 1.63 (95% CI: 1.49-1.77), respectively, compared with the nonobese group. Given that some individuals in the nonobese group were overweight, we redefined the nonobese group based on overweight status (24 kg/m^2^ ≤ BMI < 28 kg/m^2^), with the normal weight group as the reference. The aHRs were 1.15 (95% CI: 1.00-1.32) for the overweight group, 1.72 (95% CI: 1.55-1.90) for the clinical obesity group, and 1.07 (95% CI: 0.94-1.22) for the preclinical obesity group.

Compared with nonobese individuals without clinical criteria, the aHRs were 1.91 (95% CI: 1.65-2.22) for nonobese individuals with clinical criteria, 1.51 (95% CI: 1.29-1.77) for those with preclinical obesity, and 2.52 (95% CI: 2.19-2.89) for those with clinical obesity.

Compared with normal weight individuals without clinical criteria, the aHRs for HF were 1.05 (95% CI: 0.80-1.38) for overweight individuals without clinical criteria and 1.55 (95% CI: 1.29-1.85) for the preclinical obesity group (also without clinical criteria). Among individuals with clinical criteria, the aHRs were 1.90 (95% CI: 1.58-2.28) for normal weight individuals, 2.03 (95% CI: 1.67-2.46) for overweight individuals and 2.57 (95% CI: 2.19-3.01) for the clinical obesity group (Table 2).

In addition, to assess whether there is a dose–response relationship between the number of clinical criteria and the risk of HF, we analyzed the impact of 1, 2, and ≥3 clinical criteria on HF risk. Compared with the nonobese group, the aHRs for the clinical obesity group were 1.50 (95% CI: 1.37-1.64) with 1 clinical criterion, 1.91 (95% CI: 1.70-2.14) with 2 clinical criteria, and 2.20 (95% CI: 1.80-2.68) with ≥3 clinical criteria (Table 3).

**Table 3:** Associations Between Clinical Obesity and the Risk of Heart Failure

	Events/Total	Incidence Density (per 1,000 person-years)	Model 1 aHR (95% CI)	Model 2 aHR (95% CI)	Model 3 aHR (95% CI)
Nonobese	981/45,264	1.45 (1.36-1.54)	Reference	Reference	Reference
Preclinical obesity	397/18,977	1.38 (1.25-1.52)	0.95 (0.84-1.06)	0.96 (0.85-1.08)	1.00 (0.89-1.13)
Clinical criteria					
1 clinical criterion	1,171/25,199	3.24 (3.06-3.43)	1.63 (1.49-1.77)	1.55 (1.42-1.70)	1.50 (1.37-1.64)
2 clinical criteria	598/8,366	5.19 (4.79-5.62)	2.45 (2.21-2.72)	2.18 (1.95-2.43)	1.91 (1.70-2.14)
≥3 clinical criteria	133/1,325	7.86 (6.63-9.32)	3.39 (2.83-4.07)	2.75 (2.27-3.34)	2.20 (1.80-2.68)

Model 1 was adjusted for age and sex. Model 2 was further adjusted for smoking status, alcohol drinking status, physical activities, marital status, educational level, sedentary time, intake of salt, heart rate, high-sensitivity C-reactive protein, urine acid, low-density lipoprotein cholesterol, myocardial infarction, and stroke based on model 1. Model 3 was further adjusted for blood pressure–lowering agents, lipid-lowering agents, and antidiabetic agents based on model 2.

### The Relationship Between Clinical Obesity and HF Subtypes

**Figure 2:**
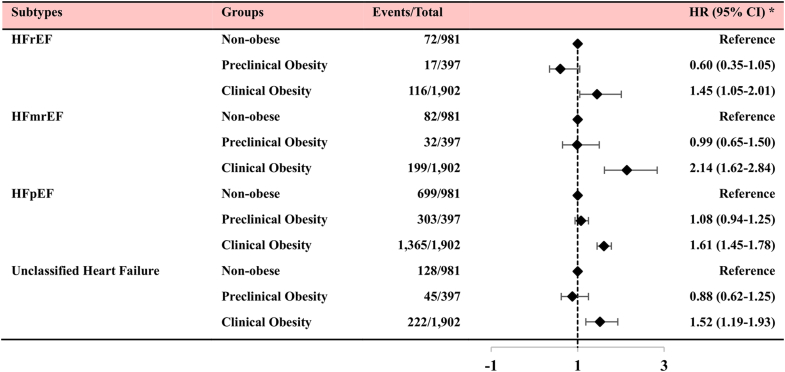
Association Between Clinical Obesity and the Risk of HF Subtypes After adjusting for covariates, compared with the nonobese group, the aHRs for the clinical obesity group in heart failure (HF) with preserved ejection fraction (HFpEF), HF with mid-range ejection fraction (HFmrEF), HF with reduced ejection fraction (HFrEF), and unclassified HF were 1.61 (95% CI: 1.45-1.78), 2.14 (95% CI: 1.62-2.84), 1.45 (95% CI: 1.05-2.01), and 1.52 (95% CI: 1.19-1.93), respectively. ∗The variables adjusted in the multivariable model included age, sex, smoking status, alcohol drinking status, physical activities, marital status, educational level, sedentary time, intake of salt, heart rate, high-sensitivity C-reactive protein, urine acid, low-density lipoprotein cholesterol, myocardial infarction, stroke, antihypertension agents, antilipidemic agents, and antidiabetic agents.

Among the HF patients, 2,367 (72.2%) were classified as HFpEF, 313 (9.5%) as HFmrEF, and 205 (6.3%) as HFrEF. Data on LVEF were missing for 395 (12.0%) cases, which were categorized as unclassified HF. After adjusting for covariates, compared with the nonobese group, the aHRs for the clinical obesity group in HFpEF, HFmrEF, HFrEF, and unclassified HF were 1.61 (95% CI: 1.45-1.78), 2.14 (95% CI: 1.62-2.84), 1.45 (95% CI: 1.05-2.01), and 1.52 (95% CI: 1.19-1.93), respectively (Figure 2).

### Sensitivity and Robustness Analysis

Sensitivity analyses were performed excluding individuals with follow-up <2 years, a history of myocardial infarction at baseline or during follow-up, or cancer. The aHRs for the clinical obesity group compared with the nonobese group were 1.68 (95% CI: 1.53-1.83), 1.64 (95% CI: 1.49-1.81), and 1.62 (95% CI: 1.48-1.77), respectively. To account for the competing risk of all-cause mortality, a competing risk model analysis was conducted which showed that the aHR for the clinical obesity group was 1.63 (95% CI: 1.49-1.78) compared with the nonobese group. These analyses confirm the robustness of the primary results (Supplemental Figure 2).

To address potential confounding, 1:1 propensity score matching was performed, including 26,648 participants in each group (clinical obesity and nonobese). The impact of clinical obesity on incident aHR is shown in Supplemental Table 8. After adjusting for all covariates, the matched analysis revealed an HR of 1.23 (95% CI: 1.18-1.28) in the clinical obesity group.

To differentiate between traditional and clinical obesity, we defined traditional obesity as BMI ≥28 kg/m^2^, with the nonobese group (BMI <24 kg/m^2^) as the reference. The aHRs were 1.22 (95% CI: 1.12-1.33) in overweight individuals and 1.76 (95% CI: 1.59-1.94) in obese individuals (Supplemental Table 9).

### Subgroup Analysis

**Figure 3:**
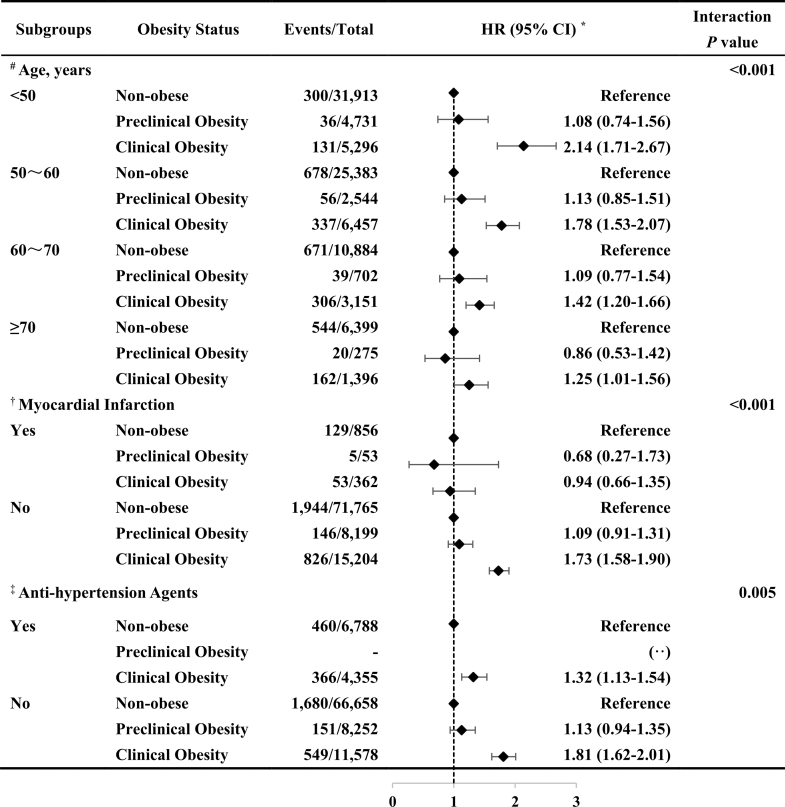
Clinical Obesity and Incident Heart Failure Across Subgroups The association between clinical obesity and heart failure was found to be dependent on age and history of myocardial infarction. The use of antihypertensive medication attenuated the risk of heart failure associated with clinical obesity. ∗The multivariable models were adjusted for age, sex, smoking status, alcohol drinking status, physical activities, marital status, educational level, sedentary time, intake of salt, heart rate, high-sensitivity C-reactive protein, urine acid, low-density lipoprotein cholesterol, myocardial infarction, stroke, blood pressure–lowering agents, lipid-lowering agents, and antidiabetic agents. ^#^Except for age. ^†^Except for myocardial infarction. ^‡^Except for blood pressure–lowering agents.

After adjusting for potential covariates, significant interactions were observed between the clinical obesity group and age, history of myocardial infarction, and antihypertensive medication use regarding the risk of HF (all, *P*_interaction_ < 0.001) (Figure 3). In the subgroup analysis, the results for individuals with a history of myocardial infarction were not consistent with the primary analysis. Compared with the nonobese group, the aHR for the clinical obesity group was 0.88 (95% CI: 0.63-1.22).

In the antihypertensive medication group, the aHR for the clinical obesity group compared with the nonobese group was 1.30 (95% CI: 1.09-1.54). In the group without antihypertensive medication, the aHR for the clinical obesity group was 1.69 (95% CI: 1.54-1.86).

We also stratified by age, dividing the study population into 4 age subgroups: <50 years, 50 to 60 years, 60 to 70 years, and ≥70 years. The results showed that compared with the nonobese group, the aHRs for the clinical obesity group in the different age subgroups were 2.14 (95% CI: 1.71-2.67), 1.78 (95% CI: 1.53-2.07), 1.42 (95% CI: 1.20-1.66), and 1.25 (95% CI: 1.01-1.56), respectively (Figure 3).

### Prognosis of HF

**Figure 4:**
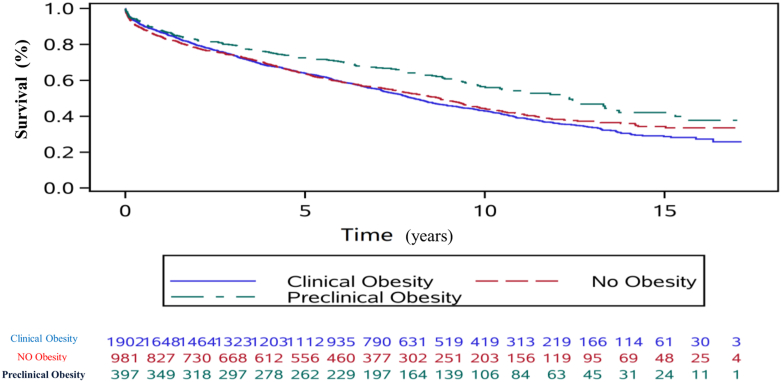
Kaplan-Meier Plots of the Survival Curves for Heart Failure Among the 3,280 patients with new-onset heart failure, all-cause mortality was considered the event of interest. The clinical obesity group exhibited the poorest prognosis, while the preclinical obesity group exhibited the best prognosis.

Among the 3,280 patients with new-onset HF, the median follow-up duration was 6.0 years (Q1-Q3: 2.2-9.4). During this period, 1,712 (52,2%) patients died. The number of all-cause deaths in the nonobese, preclinical obesity, and clinical obesity groups was 1,185 (54.0%), 51 (33.8%), and 476 (50.9%), respectively. Survival status is shown in Figure 4, with the clinical obesity group having the poorest prognosis. In this analysis, after adjusting for covariates, the aHRs for all-cause mortality were 0.77 (95% CI: 0.64-0.92) for the preclinical obesity group and 1.09 (95% CI: 0.97-1.22) for the clinical obesity group compared with the nonobese group (Table 4).

**Table 4:** Association Between Clinical Obesity and All-Cause Mortality in Patients with Heart Failure

	Deaths/Total	Model 1 aHR (95% CI)	Model 2 aHR (95% CI)	Model 3 aHR (95% CI)
Nonobese	500/981	Reference	Reference	Reference
Preclinical obesity	168/397	0.76 (0.64-0.91)	0.78 (0.65-0.93)	0.77 (0.64-0.92)
Clinical obesity	1,044/1,902	1.05 (0.95-1.17)	1.07 (0.96-1.20)	1.09 (0.97-1.22)
Stratification of the nonobese group based on the presence of clinical criteria				
Nonobese and without clinical criteria	115/272	Reference	Reference	Reference
Nonobese and with clinical criteria	385/709	1.25 (1.01-1.53)	1.15 (0.93- 1.43)	1.18 (0.95-1.47)
Preclinical obesity	168/397	0.90 (0.71- 1.13)	0.87 (0.68-1.10)	0.87 (0.68-1.10)
Clinical obesity	1,044/1,902	1.24 (1.02, 1.51)	1.19 (0.98-1.45)	1.23 (1.01-1.51)

Model 1 was adjusted for age and sex. Model 2 was further adjusted for smoking status, alcohol drinking status, physical activities, marital status, educational level, sedentary time, intake of salt, heart rate, high-sensitivity C-reactive protein, urine acid, low-density lipoprotein cholesterol, myocardial infarction, and stroke based on model 1. Model 3 was further adjusted for blood pressure–lowering agents, lipid-lowering agents, and antidiabetic agents based on model 2.

To adjust for the influence of clinical criteria on the risk of all-cause mortality in the nonobese group, we reclassified nonobese individuals with such criteria and re-analyzed the data. After adjustment for covariates, the aHRs were 1.18 (95% CI: 0.95-1.47) for the nonobese group with clinical criteria, 0.87 (95% CI: 0.68-1.10) for the preclinical obesity group, and 1.23 (95% CI: 1.01-1.51) for the clinical obesity group, each compared with the nonobese group without clinical criteria (Table 4).

Similarly, the same analysis was performed for the different HF subtypes. In the HFrEF cohort, compared with the nonobese group without clinical criteria, the aHRs for all-cause mortality were 3.04 (95% CI: 1.03-9.01) for the nonobese group with clinical criteria, 2.39 (95% CI: 0.70-8.15) for the preclinical obesity group, and 3.24 (95% CI: 1.10-9.56) for the clinical obesity group (Supplemental Table 10).

In the propensity score–matched cohort, 2,074 cases of new-onset HF were identified. The effect of clinical obesity on all-cause mortality was consistent with the primary findings and is presented in Supplemental Table 11.

Obesity was defined according to the traditional BMI criteria. The aHRs for all-cause mortality after HF were 0.92 (95% CI: 0.81-1.04) in the overweight group and 0.92 (95% CI: 0.80-1.05) in the obesity group, respectively (Supplemental Table 12).

### Exploratory Analyses

As shown in Supplemental Figure 3, two exploratory mediation analyses were conducted under alternative causal assumptions. In Pathway A, in which the clinical criterion (clinical complications) was modeled as the mediator, the proportion of the association between obesity and HF mediated through the clinical criterion was 13.8% (95% CI: 10.3%-18.2%; *P* < 0.001). In Pathway B, in which obesity was modeled as the mediator between the clinical criterion and HF, the proportion mediated was 6.2% (95% CI: 4.5%-8.4%; *P* < 0.001).

We calculated the PAR% for obesity and clinical criteria, and the results shown in Supplemental Table 13 indicate that the adjusted aHR for obesity was 1.37 (95% CI: 1.26-1.48; *P* < 0.001), with a PAR% of 16.7%. In contrast, clinical criteria, including hypertension and metabolic dysfunction, had a higher aHR of 1.75 (95% CI: 1.59-1.93; *P* < 0.001) and a PAR% of 29.3%. In post-HF patients, the PAR% for clinical criteria in relation to mortality risk was 20.8%, whereas obesity had a PAR% of 0% (Supplemental Table 14).

To explore the differences in HF risk between clinical obesity and metabolic syndrome, we further stratified the clinical obesity group into 3 subgroups: clinical obesity without metabolic syndrome, metabolic syndrome without clinical obesity, and clinical obesity with metabolic syndrome. Compared with the nonobese group, the aHRs for these subgroups were 1.54 (95% CI: 1.39-1.70), 1.41 (95% CI: 1.17-1.69), and 1.83 (95% CI: 1.65-2.02), respectively (Supplemental Table 15).

## Discussion

Based on the high-quality, complete data from this study cohort with up to 18 years of follow-up, we found that clinical obesity is an independent risk factor for new-onset HF. Even after adjusting for traditional risk factors such as smoking, inflammation levels, and a history of myocardial infarction, the association between clinical obesity and an increased risk of HF remains significant (Central Illustration). Moreover, the risk of HF rises with the number of concurrent clinical criteria. The association between clinical obesity and HF was found to be dependent on age and history of myocardial infarction. The use of antihypertensive medication attenuated the risk of HF associated with clinical obesity. Lastly, clinical obesity was not associated with an increased risk of all-cause mortality after HF.

**Central Illustration:**
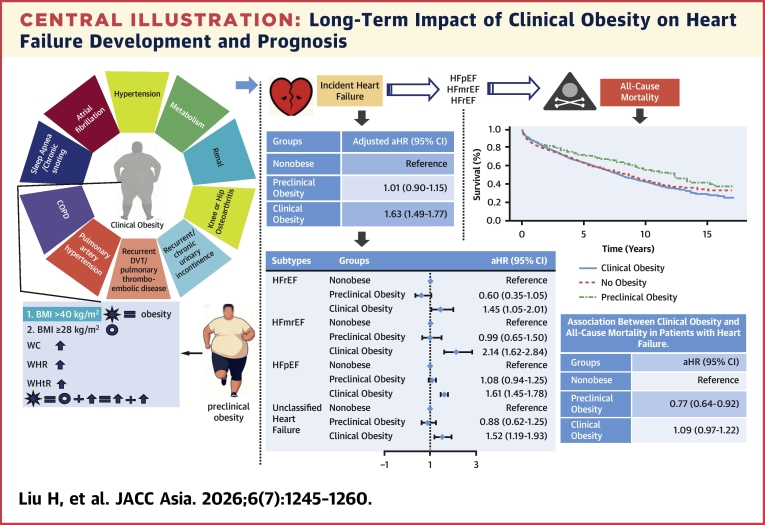
Long-Term Impact of Clinical Obesity on Heart Failure Development and Prognosis After adjusting for age, sex, smoking status, alcohol consumption, physical activity, marital status, educational level, sedentary time, salt intake, heart rate, high-sensitivity C-reactive protein, uric acid, low-density lipoprotein cholesterol, myocardial infarction, stroke, blood pressure–lowering agents, and lipid-lowering agents, clinical obesity was associated with an increased overall risk of heart failure (HF) (aHR: 1.63), with the highest risk observed in HF with mid-range ejection fraction (HFmrEF) (aHR: 2.14). Preclinical obesity showed no significant association with HF risk (aHR: 1.01). In the new-onset HF cohort, clinical obesity was not significantly associated with all-cause mortality (aHR: 1.09), whereas preclinical obesity was associated with a lower risk of mortality (aHR: 0.77). BMI = body mass index; COPD = chronic obstructive pulmonary disease; DVT = deep vein thrombosis; HFpEF = heart failure with preserved ejection fraction; HFrEF = heart failure with reduced ejection fraction; WC = waist circumference; WHR = waist-to-hip ratio; WHtR = waist-to-height ratio.

For the first time, we have shown that clinical obesity is an independent risk factor for HF, with individuals exhibiting clinical obesity showing a 63% increased risk of developing HF compared with nonobese individuals. Although previous studies have not specifically investigated the relationship between clinical obesity and incident HF, cohort studies such as Framingham, ARIC (Atherosclerosis Risk in Communities), and HUNT (Nord-Trøndelag Health Study) have shown that obese individuals (BMI ≥30 kg/m^2^) face a 70% to 150% higher relative risk of developing HF compared with those with normal weight (BMI: 18.5-25 kg/m^2^).^10^^,^^18^^,^^19^ A meta-analysis of 543,694 participants from 32 prospective cohort studies in the Asia-Pacific region revealed a 69% increased risk of HF in obese individuals compared with nonobese individuals.^11^ Studies using other anthropometric indicators (eg, waist circumference, waist-to-hip ratio, waist-to-height ratio) to define obesity have also yielded similar results.^12^^,^^18^^,^^20^, ^21^, ^22^ Compared with previous studies that assessed HF risk based on a single obesity indicator, the risk of HF associated with clinical obesity in the current study was lower than that in Western populations but similar to the risk observed in the Asia-Pacific region.

We found that the HF risk associated with clinical obesity is related to the number of organ and tissue dysfunctions related to obesity, with a dose–response relationship. As the number of obesity-related organ and tissue dysfunctions increases, the HF risk increases from 50% with 1 clinical criteria to 120% with ≥3 clinical criteria. These exploratory mediation analyses indicate that obesity may contribute to HF partly through obesity-related organ dysfunction, whereas the effect of clinical complications on HF appears largely independent of obesity. Although there is currently no direct comparison with similar studies, the overlap in definitions between clinical obesity and metabolic syndrome may explain why our findings align with the increased HF risk observed in metabolic syndrome. Studies have found that as the components of metabolic syndrome increase, the prevalence of HF increases and the risk of cardiovascular events rises.^12^^,^^23^ Moreover, previous studies have shown that the risk of cardiovascular events decreases as the number of well-controlled parameters, including blood pressure, glycemic levels, and lipid levels, increases.^13^ Other research has found that metabolic syndrome increases the risk of heart failure by 150% compared with simple obesity.^24^ In the current study, >90% of participants with clinical obesity had hypertension or metabolic dysfunction, indicating that these obesity-related clinical factors are the primary contributors to the increased risk of HF.

This exploratory analysis also found that both clinical obesity alone and metabolic syndrome are associated with an increased risk of HF, with the highest aHR observed in the subgroup with both clinical obesity and metabolic syndrome. This finding has significant clinical implications, suggesting that patients with clinical obesity require not only weight reduction but also treatment for obesity-related organ and tissue dysfunctions to mitigate the risk of HF. For instance, glucagon-like peptide-1 receptor agonists and sodium-glucose cotransporter 2 inhibitors, which have shown effectiveness in promoting weight loss, reducing blood glucose, and lowering blood pressure,^25^, ^26^, ^27^, ^28^ could be considered for treatment options for patients with clinical obesity.

Notably, 90% of patients with clinical obesity had either hypertension or metabolic dysfunction, which raises concerns about the practicality and necessity of assessing 18 clinical and organ dysfunction variables, as proposed by the Lancet Commission, that are allegedly attributable to obesity. This may pose a challenge to the implementation of the clinical obesity concept.

Age, history of myocardial infarction, and antihypertensive medication use modify the impact of clinical obesity on HF risk. Among clinical obesity individuals, the HF risk ranges from an increase of 130% in those aged <50 years to only 23% in those aged ≥70 years. This result is consistent with previous studies, which have shown that earlier exposure to HF risk factors increases the relative risk of HF events.^29^ This suggests that early intervention in younger individuals with clinical obesity may offer greater benefits. Clinical obesity did not increase the HF risk in individuals with a history of myocardial infarction, possibly due to the small sample size in the infarction group, but it cannot be ruled out that interventions have reduced the risk. Subgroup analysis indicated that antihypertensive medication mitigates HF risk in clinical obesity, which is consistent with previous studies.^30^^,^^31^

Previous studies have reported the “obesity paradox,” wherein obesity, defined by BMI, is associated with a lower mortality risk in individuals with HF.^32^^,^^33^ Our findings showed that among individuals with either overweight or obesity and HF, the aHRs and 95% CIs for all-cause mortality were both below the line of unity. However, when applying the new clinical obesity criteria, clinical obesity in patients with HF was associated with an aHR >1 for all-cause mortality (aHR: 1.09; 95% CI: 0.97-1.22). Importantly, compared with nonobese individuals without clinical criteria who developed heart failure, clinical obesity was associated with a 23% increased risk of all-cause mortality. It is important to clarify that individuals with preclinical obesity in our study met the criteria for obesity but did not have obesity-related comorbidities and therefore could be considered relatively “healthy obese.” In contrast, the nonobese reference group included some participants with multiple comorbid conditions. This raises the question of whether the observed difference in all-cause mortality risk is primarily attributable to obesity itself or to the presence of underlying diseases. We found that the presence of the obesity paradox may depend on whether the reference group includes individuals with underlying diseases. The increased risk of all-cause mortality after HF is associated with obesity-related comorbidities rather than obesity itself.

Our findings also support previous studies showing that obesity, defined by BMI, waist circumference, waist-to-hip ratio, or waist-to-height ratio, is a risk factor for HF, with a 47% to 81% increased risk. This is consistent with meta-analyses and studies conducted in the Asia-Pacific region.^11^^,^^22^^,^^34^ This result confirms that our observational cohort is representative and that there is no heterogeneity between it and other cohorts. Furthermore, clinical obesity is more strongly associated with HFmrEF than with HFpEF, which differs slightly from previous studies.^35^, ^36^, ^37^ Further research is needed to explore the relationship between clinical obesity and heart failure subtypes.

### Study Limitations

First, despite using multiple strategies, residual confounding from unmeasured factors, such as diet quality, physical activity, cardiorespiratory fitness, cardiac cachexia, nutritional status, post-HF treatments, and newer anti-obesity medications, remains possible due to the retrospective design. Clinical indicators from questionnaires or outpatient records may introduce information bias, and selection bias is inherent in this design. Nevertheless, the robustness and clinical relevance of our findings remain unaffected. Second, as an observational study, we can only show an association between clinical obesity, HF, and mortality, not causality. Third, the low proportion of female participants (20.1%) and the inclusion of 23.6% coal miners exposed to dust may limit the representativeness and generalizability of the findings. Subgroups, such as those with prior myocardial infarction, had small sample sizes, potentially limiting result stability. The lack of external validation in non-Asian populations also constrains generalizability. However, the study benefits from a large sample size (19,913 female patients), standardized health examinations, and comprehensive follow-up, enhancing internal validity and allowing a reliable assessment of obesity’s long-term impact on HF and mortality.

Fourth, in this study, clinical obesity was defined according to 10 clinical criteria and lacked diagnostic data for both the reproductive and lymphatic systems, which may have led to an underestimation of the true prevalence of clinical obesity. However, the crude prevalence of clinical obesity was 35.2%, which closely aligns with the 39.1% prevalence reported in US population data, suggesting the external validity of our findings.^38^ Fifth, although the baseline data were collected in 2006 to 2007, and changes in lifestyle, dietary habits, and health care practices may limit the applicability to current populations, the long follow-up period enhances the stability and reliability of the outcomes. Sixth, all participants had stable health insurance and routine health check-ups, which may lead to earlier detection and management of health issues, potentially underestimating the impact of clinical obesity on HF and mortality risk. However, this could also increase detection rates, thereby raising the perceived risk.

Seventh, LVEF data were missing in 395 (12.0%) of 3,280 cases, which may affect the accuracy of the subtype analyses. The absence of sufficient N-terminal pro–B-type natriuretic peptide data and challenges with time-dependent analyses due to data continuity and follow-up limitations further constrain the study. The obesity paradox observed in preclinical obesity may be influenced by reverse causation (eg, weight loss or cachexia) or selection bias. Preclinical obesity analyses were constrained by limited event numbers and follow-up duration. In addition, the inability to differentiate between cardiovascular and non-cardiovascular deaths represents another limitation. Further investigation in larger cohorts of patients newly diagnosed with HF is needed to clarify these issues.

## Conclusions

The current study confirms previous findings that obesity, as assessed by BMI, is a significant risk factor for HF. Importantly, we further show that the newly defined concept of clinical obesity is also an independent risk factor for new-onset HF. The elevated risk of HF arises not only from obesity itself but also from obesity-related organ dysfunctions. Moreover, the risk of HF increases progressively with the number of clinical criteria met, and this elevated risk extends to all-cause mortality after the onset of HF. These findings highlight the critical importance of early identification and comprehensive management of clinical obesity to prevent the development and progression of HF.

### Data Availability Statement

The Kailuan Study is not an open-access resource, and data can be obtained upon request through the corresponding author. In addition, the codebook and analytical code for this study are available upon request.

## Funding Support and Author Disclosures

The authors have reported that they have no relationships relevant to the contents of this paper to disclose.
