# Supplementary material for: Long-Term Impact of Clinical Obesity on Heart Failure Development and Prognosis: New Insights
Source: JACC Asia. 2026 May 2;6(7):1245–60. doi: 10.1016/j.jacasi.2026.01.031 (PMC13350098; doi:10.1016/j.jacasi.2026.01.031)
Supplement: Supplemental Material [file mmc1.docx]

**Appendix**

**Long-Term Impact of Clinical Obesity on Heart Failure Development and Prognosis: New Insights**

**Authors:** Hongmin Liu, MD^1#^, Tong Liu, MD, PhD^2#^, Yuntao Wu, MD, PhD^1^, Haiyan Zhao, MD, PhD^1^, Liming Lin, MD, PhD^1^, Xiang Gao, MD, PhD^3^*****, Shouling Wu, MD, PhD^1^*****

*1* *Department of Cardiology, Kailuan General Hospital, Tangshan 063001, China.*

*2 Tianjin Key Laboratory of Ionic-Molecular Function of Cardiovascular disease, Department of Cardiology, Tianjin Institute of Cardiology,* *the Second Hospital of Tianjin Medical University, Tianjin 300211, China;*

*3 Department of Nutrition and Food Hygiene, School of Public Health, Institute of Nutrition, Fudan University, Shanghai, China*

^#^Denotes joint first authors.

***Corresponding authors:** Prof. Shouling Wu, MD, PhD, Department of Cardiology, Kailuan General Hospital, 57 Xinhua East Road, Tangshan, Hebei Province, 063001, China. Email: [drwusl@163.com](mailto:drwusl@163.com), Tel: +86-315-3025655; Prof. Xiang Gao, MD, PhD, Clinical Research Institute, Fudan University, 130 Dongan Road, Shanghai 200032, China. Email: [Xiang_gao@fudan.edu.cn](mailto:Xiang_gao@fudan.edu.cn), Tel: +86-18367296319.

**Supplemental Appendix**

1. **Data Collection**
2. **Relevant Definitions**
3. **Definition of Clinical Obesity**
4. **Measurement of left ventricular ejection fraction (LVEF)**
5. **Details of Cox Proportional Hazards Models**
6. **Propensity Score Matching (PSM) Method**
7. **Definition of metabolic syndrome**

**Supplemental Appendix Table of Contents**

**Supplemental Table 1. Anthropometric Criteria for Clinical Obesity**

**Supplemental Table 2. Clinical Criteria for Clinical Obesity (Diagnosis Available Only for 2006-2007)**

**Supplemental Table 3. Chronic Disease Outpatient Diagnosis Information and Additional Diagnosis Information from Kailuan**

**Supplemental Table 4. Definitions or Stratification Criteria for Covariates.**

**Supplemental Table 5. Missing Data.**

**Supplemental Table 6. Distribution of Anthropometric Criteria in Clinical Obesity.**

**Supplemental Table 7. Distribution of Clinical Criteria.**

**Supplemental Table 8. Impact of Clinical Obesity on Incident Heart Failure: A Propensity Score Matching Analysis**

**Supplemental Table 9. Association Between Obesity and Patients with Heart Failure, n=97,267.**

**Supplemental Table 10. Clinical Obesity and All-Cause Mortality Risk in Heart Failure Subtypes: Stratification by Organ Dysfunction**

**Supplemental Table 11. Impact of Clinical Obesity on Post-HF Mortality Risk: A Propensity Score Matching Analysis**

**Supplemental Table 12. Association Between Obesity and All-Cause Mortality in Patients with Heart Failure, n=3215.**

**Supplemental Table 13. Population Attributable Risk Percentages (PAR%) for Obesity and Clinical Criteria.**

**Supplemental Table 14. Attributable Risk Analysis of Obesity and Organ Dysfunction on Post-HF Mortality Risk**

**Supplemental Table 15. Association Between Clinical Obesity, Metabolic Syndrome, and Heart Failure.**

**Supplemental Figure 1. Flowchart illustrating the study cohort and the process for assessing clinical obesit**y.

**Supplemental Figure 2. Sensitivity Analysis of the Association Between Clinical Obesity and the Risk of Incident Heart Failure.**

**Supplemental Figure 3. Mediation pathways examining the directional relationship between obesity, obesity-related clinical complications, and incident heart failure.**

1. **Data Collection**

**Anthropometric Measurements and Data Collection**

Data collection for all measurements was conducted between 07:00 and 09:00 on the day of the examination. Standardized methods were used for anthropometric measurements. Weight and height were measured simultaneously using a calibrated RGZ-120 scale, with weight recorded to the nearest 0.1 kg and height to the nearest 0.1 cm. Waist circumference was measured with a flexible tape measure in a standing position at the midpoint between the lower rib margin (subcostal margin) and the iliac crest, recorded to the nearest 0.1 cm. Hip circumference was also measured with a flexible tape measure in a standing position at the maximum circumference of the hips, at the level of the greater trochanter, recorded to the nearest 0.1 cm. All measurements were performed by trained research staff following standardized operating procedures to ensure consistency and accuracy. BMI was calculated as weight (kg) divided by height squared (m²). In line with recommendations from The Lancet Diabetes & Endocrinology Commission, the risk threshold for BMI was set at ≥28 kg/m². The waist-to-hip ratio (WHR) was calculated as waist circumference divided by hip circumference, with risk thresholds of WHR≥0·9 for men and WHR ≥0·85 for women. The waist-to-height ratio (WHtR) was calculated as waist circumference divided by height, with a risk threshold of WHtR ≥0.5.

Blood pressure was measured in the left upper arm using a calibrated mercury sphygmomanometer while the participant was seated. At least two measurements were taken after a 5-minute rest period. If the difference between the two measurements exceeded 5 mmHg, an additional reading was taken. The average of the BP measurements was used for the diagnosis of hypertension.

All participants were instructed to rest quietly for 5 minutes before electrocardiogram (ECG) measurement. A 12-lead ECG was recorded in the supine position using a Nihon Kohden ECG-9130P electrocardiograph.

**Laboratory Measurements**

Participants fasted for at least 8 hours prior to the collection of venous blood samples (5 mL) from the antecubital vein between 7:00 AM and 9:00 AM on the day of the physical examination. All biochemical analyses were conducted in a central laboratory using an automated biochemical analyzer (Hitachi 747, Hitachi Ltd., Tokyo, Japan). The measured parameters included triglycerides (TG), high-density lipoprotein cholesterol (HDL-C), low-density lipoprotein cholesterol (LDL-C), uric acid (UA), high-sensitivity C-reactive protein (hs-CRP), serum creatinine (SCr), and fasting blood glucose (FBG).

For urinary protein measurement, participants provided a random midstream morning urine sample after an 8-hour fast. Urinary protein was assessed using an automated dipstick urinalysis device. Female participants were analyzed outside of their menstrual periods. Urinary protein was recorded as semi-quantitative results: negative, trace, 1+, 2+, or 3+.

1. **Relevant Definitions**

Hypertension was defined as a systolic blood pressure (SBP) ≥140 mmHg and/or a diastolic blood pressure (DBP) ≥90 mmHg, the use of any antihypertensive medication, or a self-reported history of hypertension.

Diabetes mellitus was diagnosed according to American Diabetes Association criteria: fasting blood glucose ≥7·0 mmol/L, a documented history of diabetes, or the use of glucose-lowering medications.^1^ The estimated glomerular filtration rate (eGFR) was calculated using the Chronic Kidney Disease Epidemiology Collaboration (CKD-EPI) equation.^2^ Renal injury was defined as an eGFR <60 mL/min/1·73 m² combined with urinary protein ≥1+.

Atrial fibrillation was diagnosed based on the following ECG criteria: (1) completely irregular R-R intervals; (2) absence of distinct P waves; and (3) irregular atrial activity with the presence of fibrillatory (f) waves. The final diagnosis of atrial fibrillation is based on electrocardiographic findings during physical examinations, supplemented by a review of medical records.

1. **Definition of Clinical Obesity**

According to the standards set by The Lancet Diabetes & Endocrinology Commission, the definition of clinical obesity requires a comprehensive assessment using multidimensional data. Specifically, the criteria for obesity should be confirmed through anthropometric measurements and meet at least one clinical criteria, which includes evidence of organ/tissue dysfunction caused by obesity.

**Confirmation of anthropometric measurements should meet one of the following criteria (for detailed standards, see Supplemental Table 1):**

1. BMI > 40 kg/m².

2. BMI ≥ 28 kg/m², with at least one of the following measures elevated: waist circumference, waist-to-hip ratio, or waist-to-height ratio.

3. At least two of the following measures elevated: waist circumference, waist-to-hip ratio, or waist-to-height ratio.

| **Supplemental Table 1. Anthropometric** **Criteria for Clinical Obesity** | | |
| --- | --- | --- |
| **Anthropometric Indicator** | **Calculation Formula** | **Risk Threshold** |
| BMI, kg/m² | Weight (kg) / Height² (m²) | ≥28 |
|  |  | >40 |
| Waist Circumference, cm | ( · · ) | ≥90 (Male)  ≥85 (Female) |
| Waist-to-Height Ratio (WHtR) | Waist Circumference (cm) / Height (cm) | ≥0·50 |
| Waist-to-Hip Ratio (WHR) | Waist Circumference (cm) / Hip Circumference (cm) | ≥0·90 (Male)  ≥0·85 (Female) |

The clinical criteria include those at baseline and follow-up in the Kailuan study cohort (reordered based on the characteristics of the Kailuan study data; for detailed standards, see Supplemental Table 2):

1. Cardiovascular (Arterial): Defined by the presence of hypertension in the cohort, specifically characterized by a systolic blood pressure (SBP) ≥140 mmHg, diastolic blood pressure (DBP) ≥90 mmHg, the use of any antihypertensive medication, or a self-reported history of hypertension.
2. Metabolic: Metabolic dysfunction defined by the presence of hyperglycemia and either elevated triglyceride levels or low HDL cholesterol levels.
3. Hyperglycemia: Fasting blood glucose ≥ 6.1 mmol/L (≥ 110 mg/dL) or a diagnosis of type 2 diabetes.
4. Elevated triglycerides: Triglycerides ≥ 1.7 mmol/L (≥ 150 mg/dL).
5. Low HDL cholesterol: HDL cholesterol < 1.0 mmol/L (< 40 mg/dL).
6. Upper respiratory: Diagnosed sleep apnea or habitual snoring (Sleep apnea assessed using the STOP-Bang questionnaire for individuals who underwent health check-ups in 2014.).
7. Respiratory system: Diagnosed with chronic obstructive pulmonary disease (COPD) at discharge.
8. Cardiovascular system (Atria): Diagnosed with atrial fibrillation.
9. Cardiovascular system (Ventricles): Diagnosed with heart failure.
10. Cardiovascular (Thrombosis): Diagnosed with deep vein thrombosis and/or pulmonary embolism.
11. Renal: Estimated glomerular filtration rate (eGFR) calculated using the Chronic Kidney Disease Epidemiology Collaboration (CKD-EPI) equation. Defined as eGFR < 60 mL/min/1.73m² and urine protein ≥+ on routine urinalysis.
12. Cardiovascular system (Pulmonary): Pulmonary hypertension diagnosis obtained through the echocardiography database (pulmonary artery systolic pressure [PASP] is estimated by measuring the tricuspid regurgitation velocity on echocardiography; a PASP ≥ 40 mmHg suggests the possibility of pulmonary hypertension).
13. Chronic, severe knee or hip pain, accompanied by joint stiffness and reduced range of motion. Excludes rheumatoid arthritis, osteoarthritis, and hip replacement due to trauma.
14. Central nervous system (CNS): Diagnosed at discharge with increased intracranial pressure or recurrent headache symptoms, excluding concurrent acute cerebral infarction, cerebral hemorrhage, traumatic brain injury, and encephalitis.
15. Urinary system: Diagnosed with urinary incontinence.
16. Liver: Non-alcoholic fatty liver disease with hepatic fibrosis: Non-alcoholic fatty liver is diagnosed through liver ultrasonography and alcohol consumption history. Hepatic fibrosis is assessed using the FIB-4 score. A FIB-4 ≥ 3.25 indicates significant hepatic fibrosis or cirrhosis. The FIB-4 scoring formula is as follows:
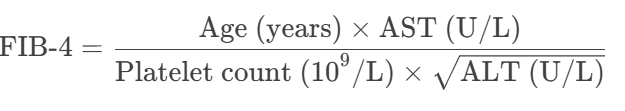

17. Activity limitations: For the 2018 health check-up data, activity limitations can be assessed using the ADL (Activities of Daily Living) questionnaire.
18. The male and female reproductive systems and lymphatic system cannot be assessed.

| **Supplemental Table 2. Clinical Criteria for Clinical Obesity (Diagnosis Available Only for 2006-2007).** | | |
| --- | --- | --- |
| **Organ, tissue, or body system** | **Diagnostic criterion (as agreed by commissioners)** | **Clinical Criteria Corresponding to the Kailuan Study** |
| **Upper airways** | **Apnoeas/hypopnoeas during sleep due to increased upper airways resistance** | **Sleep Apnea/Chronic Snoring** |
| **Respiratory** | **Hypoventilation and/or breathlessness and/or wheezing due to reduced lung and/or**  **diaphragmatic compliance** | **Chronic Obstructive Pulmonary Disease (COPD)** |
| **Cardiovascular (pulmonary)** | **Pulmonary artery hypertension** | **Pulmonary artery hypertension** |
| **Cardiovascular (ventricular)** | **Reduced Left Ventricular systolic function - Heart Failure with Reduced Ejection Fraction - HFrEF** | **Heart Failure (Regardless of Subtype)** |
| **Cardiovascular** | **Chronic fatigue, lower limb edema due to impaired diastolic dysfunction– Heart Failure with**  **Preserved Ejection Fraction - HFpEF** | **Heart Failure (Regardless of Subtype)** |
| **Cardiovascular (atrial)** | **Chronic/recurrent atrial fibrillation** | **Atrial fibrillation** |
| **Cardiovascular (thrombosis)** | **Recurrent DVT and/or pulmonary thromboembolic disease** | **Recurrent DVT and/or pulmonary thromboembolic disease** |
| **Cardiovascular (arterial)** | **Raised arterial blood pressure** | **Hypertension (①SBP>140 ② DBP>90 ③Use of Antihypertensive Medications ④History of Hypertension)** |
| **Metabolism** | **The cluster of hyperglycaemia, high triglyceride levels, and low HDL cholesterol levels** | **FBG ≥ 6.1 mmol/L or Diagnosed Diabetes Mellitus + High TG (TG ≥ 1.7 mmol/L) or Low HDL-C (HDL <1.0 mmol/L)** |
| **Liver** | **NAFLD with hepatic fibrosis** | **× （Without AST data in 2006, therefore not used）** |
| **Renal** | **Microalbuminuria with reduced eGFR** | **eGFR < 60 mL/min/1.73 m² and Proteinuria ≥ (+)** |
| **Reproductive (Male)** | **Male hypogonadism** | **×（No relevant information）** |
| **CNS** | **Signs of raised intracranial pressure such as vision loss and/or recurrent headaches** | **×（Excluding those with concurrent acute cerebral infarction, cerebral hemorrhage, traumatic brain injury, and encephalitis, data=0）** |
| **Urinary** | **Recurrent/chronic urinary incontinence** | **Recurrent/chronic urinary incontinence** |
| **Reproductive (female)** | **Anovulation, oligo-menorrhea and PCOS** | **×（No relevant information）** |
| **Musculoskeletal** | **Chronic, severe knee or hip pain associated with joint stiffness and reduced range of joint motion** | **Knee or Hip Osteoarthritis** |
| **Lymphatic** | **Lower limbs lymphedema causing chronic pain and/or reduced range of motion** | **×（No relevant information）** |
| **Limitations of day-to-day**  **activities** | **Significant, age-adjusted limitations of mobility and/or other basic Activities of Daily Living**  **(ADL=bathing, dressing, toileting, continence, eating)** | **×（No ADL questionnaire data available in 2006, therefore not used.）** |

For chronic diseases that require long-term management and cannot be completely cured, outpatient management aims to alleviate the economic burden on patients and optimize disease management strategies. To improve the completeness and accuracy of diagnostic information, this study supplements the clinical diagnostic data with information on disease diagnoses and medication use from chronic disease outpatient records.

| **Supplemental Table 3. Chronic Disease Outpatient Diagnosis Information and Kailuan Study Diagnostic Supplemental Information** | | |
| --- | --- | --- |
| Chronic Disease Outpatient Diagnosis Information | | Kailuan Study Diagnostic Supplemental Information |
| 1 | Chronic Obstructive Pulmonary Disease (COPD) | √ |
| 2 | Cor pulmonale (including: secondary cor pulmonale / primary pulmonary hypertension-related cor pulmonale, cor pulmonale (including that secondary to chronic pulmonary diseases and primary pulmonary hypertension leading to cor pulmonale)) | √ |
| 3 | Systemic Lupus Erythematosus (SLE) (including: Systemic Lupus Erythematosus / Systemic Lupus Erythematosus with complications) | √ |
| 4 | Rheumatoid Arthritis (including: Rheumatoid Arthritis / Rheumatoid Arthritis with severe physical disability / Rheumatoid Arthritis with severe physical disability or severe organ damage / Rheumatoid Arthritis with functional impairment / Rheumatoid Arthritis in the active phase) | √ |
| 5 | Viral Hepatitis (including: Viral Hepatitis / Chronic Hepatitis in the active phase / Chronic moderate to severe viral hepatitis / Chronic Hepatitis B / Chronic Hepatitis C) | √ |
| 6 | Diabetic Nephropathy | √ |
| 7 | Hypertensive Nephropathy | √ |
| 8 | Cerebral Infarction |  |
| 9 | Coronary Artery Disease |  |
| 10 | **Cirrhosis** |  |
| 11 | **Peripheral Vascular Disease** |  |
| 12 | **Parkinson's Disease** |  |
| 13 | Myasthenia Gravis |  |
| 14 | **Peptic Ulcer Disease** |  |
| 15 | Chronic Atrophic Gastritis |  |
| 16 | **Ulcerative Colitis** |  |
| 17 | **Psychiatric Disorders** |  |
| 18 | **Cerebral and Somatic Organic Diseases** |  |
| 19 | Uremia |  |
| 20 | Post-Organ Transplantation Treatment |  |
| 21 | Malignant Tumors |  |
| 22 | Aplastic Anemia |  |
| 23 | Leukemia |  |
| 24 | Motor Neuron Disease |  |
| 25 | Myelodysplastic Syndromes (MDS) |  |
| 26 | Hemophilia |  |
| The following are the diseases newly included in 2022: | | |
| 27 | Sjögren's Syndrome |  |
| 28 | **Autoimmune Hepatitis** |  |
| 29 | **Post-Cardiac Valve Replacement Treatment** |  |
| 30 | **Heart Failure** |  |
| 31 | **Cardiomyopathy** |  |
| 32 | **Arrhythmias** |  |
| 33 | Arterial Embolism |  |
| 34 | Atherosclerotic Occlusive Disease |  |
| 35 | Psoriasis |  |
| 36 | **Scleroderma** |  |
| 37 | Atopic Dermatitis |  |
| 38 | **Kashin-Beck Disease** |  |
| 39 | Keshan Disease |  |
| 40 | Phenylketonuria (PKU) |  |
| 41 | **Gaucher Disease** |  |
| 42 | Tuberous Sclerosis |  |
| 43 | Glaucoma |  |
| 44 | Rheumatic Heart Disease |  |
| 45 | Behçet's Disease |  |
| 46 | Rheumatoid Polymyalgia |  |
| 47 | Dermatomyositis |  |
| 48 | Systemic Scleroderma |  |
| 49 | Polymyositis |  |
| 50 | Spondyloarthritis |  |
| 51 | Brucellosis |  |
| 52 | Short Bowel Syndrome |  |
| 53 | Chronic Pancreatitis |  |
| 54 | Non-viral Hepatitis Type - Hepatolenticular Degeneration |  |
| 55 | Congenital Heart Disease in Children |  |
| 56 | **Chronic Venous Insufficiency of the Lower Limbs** |  |
| 57 | Post Pacemaker Implantation |  |
| 58 | Alzheimer's Disease |  |
| 59 | Epilepsy |  |
| 60 | Multiple Sclerosis |  |
| 61 | Moyamoya Disease |  |
| 62 | Post-intracranial/extracranial Vascular Stenting |  |
| 63 | Post-subarachnoid Hemorrhage Sequelae |  |
| 64 | Cerebral Palsy |  |
| 65 | Progressive Muscular Dystrophy |  |
| 66 | Cerebrovascular Disease |  |
| 67 | **Other Neurological Disorders** |  |
| 68 | **Borderline Tumor Outpatient Treatment** |  |
| 69 | **Hematologic and Hemopoietic Organ Disorders Involving Immune Mechanisms** |  |
| 70 | Purpura |  |
| 71 | Multiple Myeloma |  |
| 72 | Primary Thrombocythemia |  |
| 73 | Polycythemia Vera |  |
| 74 | Langerhans Cell Histiocytosis |  |
| 75 | Chronic Active Epstein-Barr Virus Infection |  |
| 76 | Chronic Nephritis (including: Chronic Glomerulonephritis / Chronic Nephritis / Chronic Kidney Disease CKD Stage 3-4) |  |
| 77 | **Nephrotic Syndrome** |  |
| 78 | Myelofibrosis |  |
| 79 | **Syringomyelia** |  |
| 80 | Avascular Necrosis |  |
| 81 | Chronic Osteomyelitis |  |
| 82 | AIDS |  |
| 83 | Osteoporosis |  |
| 84 | Lumbar Disc Herniation |  |
| 85 | Interstitial Lung Disease |  |
| 86 | Tuberculosis |  |
| 87 | Bronchiectasis |  |
| 88 | Bronchial Asthma |  |
| 89 | New Pulmonary Dysfunction |  |
| 90 | Gout |  |
| 91 | Thyroid Dysfunction |  |
| 92 | Other Endocrine and Metabolic Disorders |  |
| 93 | Spinal Canal Stenosis |  |

1. **Measurement of left ventricular ejection fraction (LVEF)**

We used the PHILIPS IE33 and EPIQ 7C color Doppler ultrasound machines, equipped with a 1-5 MHz S5-1 probe, to measure the Left Ventricular Diameter at End-Diastole (LVDd) and Left Ventricular Diameter at End-Systole (LVDs) via M-mode echocardiography. LVEF was calculated using the modified Teichholtz formula, as follows:

LVEF=$\frac{EDV-ESV}{EDV}\times100\%$

In cases with segmental wall motion abnormalities, the biplane Simpson method was used to measure LVEF.

1. **Details of Cox Proportional Hazards Models**

We used Cox proportional hazards models to examine the effect of clinical obesity on the incidence of new-onset HF, with non-obese individuals as the reference group: Model 1: Adjusted for age and sex. Model 2: Adjusted for age (continuous), sex, smoking status, alcohol consumption, physical activity, marital status, educational level, sedentary behavior, salt intake, heart rate (HR, continuous), high-sensitivity C-reactive protein (hs-CRP, continuous), low-density lipoprotein cholesterol (LDL-C, <3.4 vs ≥3.4 mmol/L), hyperuricemia (yes/no), history of myocardial infarction (MI, yes/no), and history of cerebrovascular accident (stroke, yes/no).Model 3: Further adjusted for the use of antihypertensive medications (yes/no), lipid-lowering medications (yes/no), and antidiabetic medications (yes/no), in addition to the variables in Model 2. These models were used to analyze the impact of clinical obesity on the risk of new-onset HF while accounting for various demographic, clinical history, and treatment-related variables.

For the Cox regression models, the proportional hazards assumption (PH test) for all covariates was assessed using maximum likelihood estimation. For covariates that did not meet the assumption, we corrected for this by including interaction terms between these covariates and the log of time (log(t)) in the model.

1. Propensity Score Matching (PSM) Method

The propensity scores were estimated using logistic regression, including all covariates: age, gender, smoking status, alcohol consumption, physical activity, marital status, educational level, sedentary time, salt intake, heart rate, high-sensitivity C-reactive protein, uric acid, low-density lipoprotein cholesterol, myocardial infarction, stroke, use of blood pressure-lowering agents, lipid-lowering agents, and anti-diabetic agents. Matching was performed using a 1:1 nearest-neighbor matching algorithm with a caliper of 0.2 times the standard deviation of the logit of the propensity score. After matching, standardized mean differences (SMD) were calculated for each baseline characteristic, with a threshold of 0.1 applied to ensure balance between the two groups.

1. **Definition of metabolic syndrome**

Metabolic syndrome refers to a cluster of metabolic abnormalities that elevate the risk of cardiovascular disease. These conditions include:

**Abdominal obesity** – Excess visceral fat, typically assessed by waist circumference: ≥90 cm in men, ≥85 cm in women.

**Hypertension** – A blood pressure measurement of ≥130/85 mmHg, or a prior diagnosis of hypertension.

**Hyperglycemia** – Elevated fasting glucose levels, defined as ≥6.0 mmol/L, or a diagnosis of diabetes mellitus.

**Hypertriglyceridemia** – Elevated triglyceride levels, typically ≥1.7 mmol/L.

**Low HDL cholesterol** – Reduced high-density lipoprotein (HDL) cholesterol, generally <1.04 mmol/L.

A diagnosis of metabolic syndrome is made when at least three of these criteria are met.

| **Supplemental Table 4. Definitions or Stratification Criteria for Covariates.** | |
| --- | --- |
| Covariates | Definitions or Stratification Criteria |
| Smoking Status | ①Never Smoked  ②Former Smoker  ③Occasional Smoker  ④Daily Smoker  Daily smoking is defined as smoking at least one cigarette per day on average over the past year, with individuals who have quit smoking for less than one year also classified as smokers. Occasional smoking is defined as smoking fewer than one cigarette per day on average. |
| Drinking Status | ①Currently Non-drinker  ②Former or Occasional Drinker (1-3 times per month)  ③Frequent Drinker (at least once per day) |
| Physical activity | ①No Physical Activity  ②Occasional Physical Activity  ③Regular Physical Activity (More than 80 minutes per week) |
| Marital status | ①Never Married  ②Married  ③Divorced  ④Widowed  ⑤Remarried |
| Educational Level | ①No Formal Education  ②Primary School  ③Middle School  ④High School  ⑤College or Higher |
| Sedentary Time | ①Daily Sitting Time < 4 Hours  ②Daily Sitting Time 4-8 Hours  ③Daily Sitting Time > 8 Hours |
| Salt Intake | ①Low Preference (<6g per day)  ②Moderate Preference (6-12g per day)  ③High Preference (>12g per day) |
| Hyperuricemia | ①Men: Serum uric acid level > 420 μmol/L (7.0 mg/dL)  ②Women: Serum uric acid level > 360 μmol/L (6.0 mg/dL) |
| Low-Density Lipoprotein Cholesterol (LDL-C) | **①LDL-C <3·4 mmol/L**  **②LDL-C≥3·4 mmol/L** |

| **Supplemental Table 5. Missing Data.** | | |
| --- | --- | --- |
| Variables | Total | Missing Data |
| Smoking Status | 99,131 | 2082 (2.10%) |
| Drinking Status | 99,131 | 2053 (2.07%) |
| Physical activity | 99,131 | 2968 (2.99%) |
| Marital status | 99,131 | 2727 (2.75%) |
| Educational Level | 99,131 | 2768 (2.79%) |
| Sedentary Time | 99,131 | 3049 (3.08%) |
| Intake of Salt | 99,131 | 2907 (2.93%) |
| Heart Rate | 99,131 | 4521 (4.56%) |
| Urine Acid | 99,131 | 604 (0.61%) |
| BMI | 99,131 | 75 (0.08%) |
| Weight | 99,131 | 44 (0.04%) |
| Height | 99,131 | 40 (0.04%) |
| Hip Circumference | 99,131 | 931 (0.94%) |
| Waist-to-hip ratio | 99,131 | 931 (0.94%) |
| Waist-to-height ratio | 99,131 | 41 (0.04%) |
| Systolic Blood Pressure | 99,131 | 369 (0.37%) |
| Diastolic Blood Pressure | 99,131 | 369 (0.37%) |
| eGFR, mL/min/1·73 m2 | 99,131 | 1013 (1.02%) |
| Fasting Blood Glucose | 99,131 | 567 (0.57%) |
| hs-CRP | 99,131 | 2008 (2.03%) |
| LDL-C | 99,131 | 648 (0.65%) |
| HDL-C | 99,131 | 545 (0.55%) |
| Triglycerides | 99,131 | 567 (0.57%) |
| Myocardial Infarction | 99,131 | 2692 (2.72%) |
| Stroke | 99,131 | 2701 (2.72%) |
| Diabetes | 99,131 | 10 (0.01%) |
| Anti-diabetic Agents | 99,131 | 2439 (2.46%) |
| Anti-lipidemic Agents | 99,131 | 1800 (1.82%) |
| Anti-hypertension Agents | 99,131 | 1500 (1.51%) |
| *Abbreviation*: hs-CRP, high-sensitivity C-reactive protein; eGFR, estimated glomerular filtration rate; HDL-C, high-density lipoprotein cholesterol; LDL-C, low-density lipoprotein cholesterol; BMI, body mass index. | | |

**Supplemental Table 6. Distribution of Anthropometric Criteria in Clinical Obesity.**

| Obesity Risk Threshold | Total  (N=99,131) | Non-obese  (N=45,264) | Preclinical Obesity  (N=18,977) | Clinical Obesity  (N=34,890) |
| --- | --- | --- | --- | --- |
| BMI>40 kg/m², (cases) | 85 | 0 | 26 | 59 |
| 28 kg/m²≤BMI≤40 kg/m², (cases) | 18,316 | 757 | 4,612 | 12,947 |
| Waist circumference≥90cm (Male) or≥85cm (Female), (cases) | 42,276 | 95 | 14,136 | 28,045 |
| Waist-to-hip ratio≥0.90 (Male) or ≥0.85 (Female), (cases) | 50,871 | 7,813 | 15,496 | 27,562 |
| Waist-to-height ratio≥0.50, (cases) | 63,216 | 9,819 | 18,796 | 34,601 |

| **Supplemental Table 7. Distribution of Clinical Criteria.** | | | | |
| --- | --- | --- | --- | --- |
| **Clinical Criteria** | **Total**  **(N=99,131)** | **Non-Obese**  **(N=45,264)** | **Preclinical Obesity (N=18,977)** | **Clinical Obesity**  **(N=34,890)** |
| One Clinical Criteria | 41,563 (41.93%) | 16,364 (36.15%) | 0 | 25,199 (72.22%) |
| Two Clinical Criteria | 11,699 (11.80%) | 3,333 (7.36%) | 0 | 8,366 (23.98%) |
| Three Clinical Criteria | 1,508 (1.52%) | 270 (0.60%) | 0 | 1,238 (3.55%) |
| Four Clinical Criteria | 93 (0.09%) | 9 (0.02%) | 0 | 84 (0.24%) |
| Five Clinical Criteria | 3 (0.00%) | 0 | 0 | 3 (0.01%) |
| Hypertension | 45,350 (45.75%) | 15,986 (35.32%) | 0 | 29,364 (84.16%) |
| Metabolism | 7,966 (8.04%) | 1,944 (4.29%) | 0 | 6,022 (17.26%) |
| Upper airways (Snore) | 13,680 (13.80%) | 4,941 (10.92%) | 0 | 8,739 (20.05%) |
| Upper airways (Obstructive Sleep Apnea) | 30 (0.03%) | 4 (0.01%) | 0 | 26 (0.07%) |
| Respiratory (COPD) | 1,311 (1.32%) | 494 (1.09%) | 0 | 817 (2.34%) |
| Renal | 1,009 (1.02%) | 341 (0.75%) | 0 | 668 (1.91%) |
| Atrial Fibrillation | 429 (0.43%) | 129 (0.28%) | 0 | 300 (0.86%) |
| Pulmonary Artery Hypertension | 84 (0.08%) | 31 (0.07%) | 0 | 53 (0.15%) |
| Musculoskeletal | 8 (0.01%) | 4 (0.01%) | 0 | 4 (0.01%) |
| Recurrent DVT and/or Pulmonary Thromboembolic Disease | 17 (0.02%) | 5 (0.01%) | 0 | 12 (0.03%) |
| Recurrent/chronic Urinary Incontinence | 1 (0.00%) | 0 | 0 | 1 (0.00%) |

| **Supplemental Table 8. Impact of Clinical Obesity on Incident Heart Failure: A Propensity Score Matching Analysis** | | | | |
| --- | --- | --- | --- | --- |
| **Obesity Status** | **HF/Total** | **Model 1**  **HR (95% CI)** | **Model 2**  **HR (95% CI)** | **Model 3**  **HR (95% CI)** |
| Non-Obese | 815/26,648 | Reference | Reference | Reference |
| clinical Obesity | 1,259/26,648 | 1.26 (1.20-1.32) | 1.25 (1.19-1.30) | 1.23 (1.18-1.28) |
| Model 1 was adjusted for age and gender.  Model 2 was further adjusted for smoking status, alcohol drinking status, physical activities, marital status, educational level, sedentary time, intake of salt, heart rate, high-sensitivity C-reactive protein, urine acid, low-density lipoprotein cholesterol, myocardial infarction, and stroke based on model 1.  Model 3 was further adjusted for blood pressure lowering agents, lipid-lowering agents, and anti-diabetic agents based on model 2.  HR=Hazard Ratio. | | | | |

| **Supplemental Table 9. Association Between Obesity and Patients with Heart Failure, n=97,267.** | | |
| --- | --- | --- |
| **BMI, kg/m^2^** | **Deaths/Total** | **Model A**  **HR (95% CI)** |
| 18.5≤BMI<24 | 924/37,363 | Reference |
| 24≤BMI<28 | 1,392/41,503 | 1.22 (1.12-1.33) |
| BMI≥28 | 899/18,401 | 1.76 (1.59-1.94) |
| **Obesity Classification Based on BMI According to WHO Standards** | | |
| 18.5≤BMI<25 | 1,256/49,255 | Reference |
| 24≤BMI<30 | 1,558/40,088 | 1.40 (1.29-1.51) |
| BMI≥30 | 401/7,924 | 1.80 (1.59-2.03) |
| Model A was adjusted for age, gender, smoking status, alcohol drinking status, physical activities, marital status, educational level, sedentary time, intake of salt, heart rate, high-sensitivity C-reactive protein, urine acid, low-density lipoprotein cholesterol, myocardial infarction, stroke, anti-hypertension agents, anti-lipidemic agents, and anti-diabetic agents. WHO =World Health Organization; HR=Hazard Ratio. | | |

| **Supplemental Table 10. Clinical Obesity and All-Cause Mortality Risk in Heart Failure Subtypes: Stratification by Organ Dysfunction** | | | | |
| --- | --- | --- | --- | --- |
| **Obesity Status** | **Non-Obese and without Clinical Criteria** | **Non-Obese and with Clinical Criteria** | **Preclinical Obesity** | **Clinical Obesity** |
| **HFpEF** |  |  |  |  |
| Deaths/Total | 89/202 | 243/497 | 121/303 | 685/1365 |
| Model 1 | Reference | 1.02 (0.80-1.30) | 0.76 (0.58-0.99) | 1.01 (0.81-1.26) |
| Model 2 | Reference | 0.92 (0.72-1.18) | 0.74 (0.56-0.98) | 0.97 (0.78-1.22) |
| Model 3 | Reference | 0.95 (0.74-1.22) | 0.75 (0.57-0.98) | 1.00 (0.79-1.26) |
| **HFmrEF** |  |  |  |  |
| Deaths/Total | 11/27 | 38/55 | 17/32 | 141/199 |
| Model 1 | Reference | 1.76 (0.89-3.46) | 1.39 (0.65-2.97) | 1.79 (0.97-3.32) |
| Model 2 | Reference | 1.79 (0.85-3.74) | 1.46 (0.65-3.27) | 1.85 (0.95-3.64) |
| Model 3 | Reference | 1.78 (0.85-3.75) | 1.42 (0.63-3.20) | 1.83 (0.92-3.64) |
| **HFrEF** |  |  |  |  |
| Deaths/Total | 4/15 | 40/57 | 9/17 | 87/116 |
| Model 1 | Reference | 3.76 (1.33-10.61) | 2.64 (0.81-8.59) | 3.71 (1.35-10.18) |
| Model 2 | Reference | 3.14 (1.07-9.23) | 2.37 (0.69-8.11) | 3.43 (1.18-9.94) |
| Model 3 | Reference | 3.04 (1.03-9.01) | 2.39 (0.70-8.15) | 3.24 (1.10-9.56) |
| **Unclassified Heart Failure** |  |  |  |  |
| Deaths/Total | 11/28 | 64/100 | 21/45 | 131/222 |
| Model 1 | Reference | 1.95 (1.03-3.69) | 1.19 (0.57-2.46) | 1.61 (0.87-2.98) |
| Model 2 | Reference | 2.13 (1.09-4.17) | 1.23 (0.58-2.61) | 1.65 (0.87-3.14) |
| Model 3 | Reference | 2.13 (1.08-4.18) | 1.22 (0.58-2.59) | 1.64 (0.85-3.15) |
| Model 1 was adjusted for age and gender.  Model 2 was further adjusted for smoking status, alcohol drinking status, physical activities, marital status, educational level, sedentary time, intake of salt, heart rate, high-sensitivity C-reactive protein, urine acid, low-density lipoprotein cholesterol, myocardial infarction, and stroke based on model 1.  Model 3 was further adjusted for blood pressure lowering agents, lipid-lowering agents, and anti-diabetic agents based on model 2. | | | | |

| **Supplemental Table 11. Impact of Clinical Obesity on Post-HF Mortality Risk: A Propensity Score Matching Analysis** | | | | |
| --- | --- | --- | --- | --- |
| **Obesity Status** | **death/HF** | **Model 1**  **HR (95% CI)** | **Model 2**  **HR (95% CI)** | **Model 3**  **HR (95% CI)** |
| Non-Obese | 432/815 | Reference | Reference | Reference |
| clinical Obesity | 669/1,259 | 1.02 (0.91-1.16) | 1.03 (0.91-1.16) | 1.03 (0.91-1.17) |
| Model 1 was adjusted for age and gender.  Model 2 was further adjusted for smoking status, alcohol drinking status, physical activities, marital status, educational level, sedentary time, intake of salt, heart rate, high-sensitivity C-reactive protein, urine acid, low-density lipoprotein cholesterol, myocardial infarction, and stroke based on model 1.  Model 3 was further adjusted for blood pressure lowering agents, lipid-lowering agents, and anti-diabetic agents based on model 2.  HR=Hazard Ratio. | | | | |

| **Supplemental Table 12. Association Between Obesity and All-Cause Mortality in Patients with Heart Failure, n=3215.** | | | | |
| --- | --- | --- | --- | --- |
| **BMI, kg/m^2^** | **Deaths/Total** | **Model 1**  **HR (95% CI)** | **Model 2**  **HR (95% CI)** | **Model 3**  **HR (95% CI)** |
| 18.5≤BMI<24 | 526/924 | Reference | Reference | Reference |
| 24≤BMI<28 | 697/1,392 | 0.87 (0.78-0.98) | 0.92 (0.81-1.03) | 0.92 (0.81-1.04) |
| BMI≥28 | 446/899 | 0.90 (0.79-1.02) | 0.91 (0.79-1.04) | 0.92 (0.80-1.05) |
| **Obesity Classification Based on BMI According to WHO Standards** | | | | |
| 18.5≤BMI<25 | 682/1,256 | Reference | Reference | Reference |
| 25≤BMI<30 | 801/1,558 | 0.95 (0.86-1.05) | 0.98 (0.88-1.10) | 0.99 (0.89-1.11) |
| BMI≥30 | 186/401 | 0.86 (0.73-1.00) | 0.84 (0.70-1.00) | 0.86 (0.72-1.03) |
| Model 1 was adjusted for age and gender.  Model 2 was further adjusted for smoking status, alcohol drinking status, physical activities, marital status, educational level, sedentary time, intake of salt, heart rate, high-sensitivity C-reactive protein, urine acid, low-density lipoprotein cholesterol, myocardial infarction, and stroke based on model 1.  Model 3 was further adjusted for anti-hypertension agents, anti-lipidemic agents, and anti-diabetic agents based on model 2. HR=Hazard Ratio. | | | | |

| **Supplemental Table 13. Population Attributable Risk Percentages (PAR%) for Obesity and Clinical Criteria in Heart Failure Risk.** | | | | |
| --- | --- | --- | --- | --- |
| **Risk Factor** | **Sample Size /Total** | **Population Proportion** | **Model**  **HR (95% CI)** | **PAR%** |
| Non-obesity | 45,264 /99,131 | 45.7% | Reference | - |
| Obesity | 53,867 /99,131 | 54.3% | 1.37 (1.26-1.48) | 16.7% |
| Without clinical criteria | 44,265/99,131 | 44.6% | Reference | - |
| clinical criteria | 54,866/99,131 | 55.4% | 1.75 (1.59-1.93) | 29.3% |
| Model was adjusted for age and gender, smoking status, alcohol drinking status, physical activities, marital status, educational level, sedentary time, intake of salt, heart rate, high-sensitivity C-reactive protein, urine acid, low-density lipoprotein cholesterol, myocardial infarction, stroke, blood pressure lowering agents, lipid-lowering agents, and anti-diabetic agents. | | | | |

| **Supplemental Table 14. Attributable Risk Analysis of Obesity and Organ Dysfunction on Post-HF Mortality Risk** | | | | |
| --- | --- | --- | --- | --- |
| **Obesity Status** | **Cases/Total** | **Population Proportion** | **Model**  **HR (95% CI)** | **PAR%** |
| Non-obese | 981/3,280 | 29.9% | Reference | - |
| Obesity | 2,299/3,280 | 70.1% | 1.00 (0.90-1.12) | 0 |
| Without clinical criteria | 669/3,280 | 20.4% | Reference | - |
| With clinical criteria | 2,611/3,280 | 79.6% | 1.33 (1.16-1.52) | 20.8% |
| Model was adjusted for age and gender, smoking status, alcohol drinking status, physical activities, marital status, educational level, sedentary time, intake of salt, heart rate, high-sensitivity C-reactive protein, urine acid, low-density lipoprotein cholesterol, myocardial infarction, stroke, blood pressure lowering agents, lipid-lowering agents, and anti-diabetic agents. | | | | |

| **Supplemental Table 15. Association Between Clinical Obesity, Metabolic Syndrome, and Heart Failure.** | | | | |
| --- | --- | --- | --- | --- |
| **Obesity Status** | **HF/Total** | **Model 1**  **HR (95% CI)** | **Model 2**  **HR (95% CI)** | **Model 3**  **HR (95% CI)** |
| Non-Obese | 900/43,416 | Reference | Reference | Reference |
| Preclinical Obesity | 333/16,688 | 0.94 (0.83-1.07) | 0.96 (0.85-1.10) | 1.01 (0.89-1.15) |
| clinical Obesity without MS | 807/17,265 | 1.63 (1.48-1.79) | 1.59 (1.44-1.76) | 1.54 (1.39-1.70) |
| MS without clinical Obesity | 145/4,137 | 1.54 (1.29-1.83) | 1.46 (1.21-1.75) | 1.41 (1.17-1.69) |
| clinical Obesity and MS | 1,095/17,625 | 2.32 (2.12-2.54) | 2.07 (1.88-2.28) | 1.83 (1.65-2.02) |
| Model 1 was adjusted for age and gender.  Model 2 was further adjusted for smoking status, alcohol drinking status, physical activities, marital status, educational level, sedentary time, intake of salt, heart rate, high-sensitivity C-reactive protein, urine acid, low-density lipoprotein cholesterol, myocardial infarction, and stroke based on model 1.  Model 3 was further adjusted for blood pressure lowering agents, lipid-lowering agents, and anti-diabetic agents based on model 2.  HR=Hazard Ratio. | | | | |

**Supplemental Figure 1. Flowchart illustrating the study cohort and the process for**

**assessing clinical obesity.**

NO

99,131 participants included in the statistical analysis.

Clinical confirmation of abnormal fat mass by BMI, waist circumference, waist-to-hip ratio and Waist-to-Height Ratio.

No obesity

(normal fat mass)

(n=45,264)

Medical history and physical examination

• No limitations of daily activities

• No signs or symptoms

• Signs or symptoms

• Organ dysfunction

Limitations of daily activities

Preclinical obesity

(n=18,977)

Clinical obesity

(n=34,890)

Further diagnostic assessment

Obesity related

Not obesity related

Yes

Participants underwent physical examinations between 2006 and 2007 and met the inclusion criteria for this study (n=99,367)

139 participants were excluded due to conditions such as Cushing's syndrome, glomerulonephritis, rheumatoid arthritis, systemic lupus erythematosus, and Sjögren's syndrome.

97 participants with a history of heart failure were also excluded.

|  |
| --- |


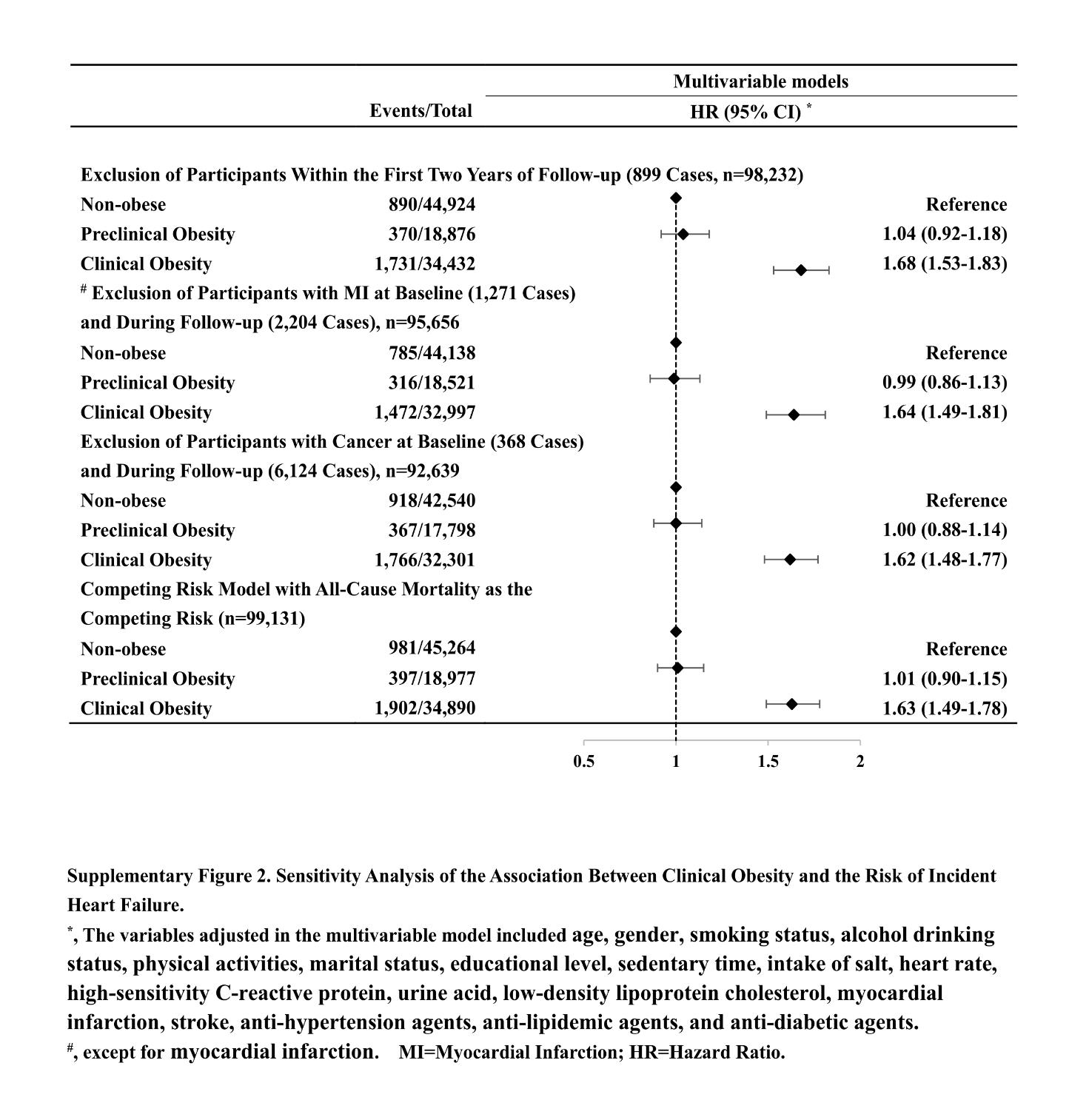


Supplemental Figure 3. Mediation pathways examining the directional relationship between obesity, obesity-related clinical complications, and incident heart failure. Pathway A: clinical complications as the mediator between obesity and HF. Pathway B: obesity as the mediator between clinical complications and HF. All models were adjusted for age, sex, smoking status, alcohol consumption, physical activity, marital status, educational level, sedentary time, salt intake, heart rate, high-sensitivity C-reactive protein, uric acid, low-density lipoprotein cholesterol, history of myocardial infarction and stroke, and the use of antihypertensive, lipid-lowering, and antidiabetic medications.

Abbreviations: NDE, natural direct effect; HR, Hazard Ratio; CI, confidence interval.

**Obesity**

**clinical criterion**

**(clinical complications)**

**Heart failure**

Proportion mediated: 13.8%

（95%CI, 10.3%-18.2%), p<0.001

NDE HR:1.37, 95%CI: 1.26, 1.48, *p*<0.001

Total effect HR:1.43, 95%CI: 1.26, 1.48, *p*<0.001

**clinical criterion**

**(clinical complications)**

**Obesity**

**Heart failure**

NDE HR:1.75, 95%CI: 1.59, 1.93, *p*<0.001

Total effect HR:1.82, 95%CI: 1.65, 2.00, *p*<0.001

Proportion mediated: 6.2%

（95%CI, 4.5%-8.4%), p<0.001

A

B

REFERENCES

1. ElSayed NA, Aleppo G, Aroda VR, et al. 2. Classification and Diagnosis of Diabetes: Standards of Care in Diabetes-2023. *Diabetes Care* 2023; **46**(Suppl 1): S19-s40.

2. Levey AS, Stevens LA, Schmid CH, et al. A new equation to estimate glomerular filtration rate. *Ann Intern Med* 2009; **150**(9): 604-12.
